# Fluorine effect in nucleophilic fluorination at C4 of 1,6-anhydro-2,3-dideoxy-2,3-difluoro-β-D-hexopyranose

**DOI:** 10.3762/bjoc.16.237

**Published:** 2020-11-25

**Authors:** Danny Lainé, Vincent Denavit, Olivier Lessard, Laurie Carrier, Charles-Émile Fecteau, Paul A Johnson, Denis Giguère

**Affiliations:** 1 Département de chimie, Université Laval, 1045 av. De la Médecine, Québec City, Qc, G1V 0A6, Canada

**Keywords:** deoxyfluorination, Et_3_N·3HF, fluorine effect, polyfluorinated carbohydrates, polyfluoroalditol analogues

## Abstract

In this work, we have developed a simple synthetic approach using Et_3_N·3HF as an alternative to the DAST reagent. We controlled the stereochemistry of the nucleophilic fluorination at C4 of 1,6-anhydro-2,3-dideoxy-2,3-difluoro-4-*O*-triflate-β-ᴅ-talopyranose using Et_3_N·3HF or in situ generated Et_3_N·1HF. The influence of the fluorine atom at C2 on reactivity at C4 could contribute to a new fluorine effect in nucleophilic substitution. Finally, with the continuous objective of synthesizing novel multi-vicinal fluorosugars, we prepared one difluorinated and one trifluorinated alditol analogue.

## Introduction

The biological significance of carbohydrates includes, but are not limited to, immune regulation, infection, and cancer metastasis. Research in the field of molecular biology allowed the discovery of glycomimetics to study various biochemical processes [1]. Therefore, the use of bioisosteres of carbohydrates functional groups is a popular approach in glycobiology [2]. As such, the synthesis of fluorosugars, including polyfluorinated analogues, is an interesting strategy to study biological systems [3–7]. The replacement of OH groups by fluorine atoms arise from their similarities in term of polarity and isosteric relationship [3,8]. For a long time, fluorinated carbohydrates have been used as a method to stabilize glycosidic bonds [9–11] or for epitope mapping [12–13]. More recently, heavily fluorinated carbohydrates were involved, among other things [5], in binding with UDP-Gal mutase [14], immunoglobulin [15–16], and glycogen phosphorylase [17].

**Figure 1:**
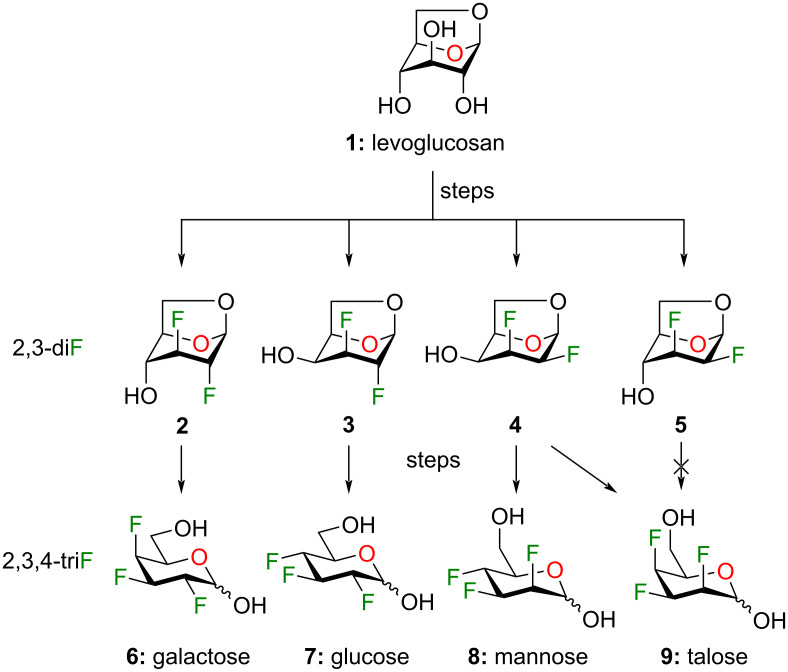
Previously described synthesis of 2,3,4-trifluorinated analogues of galactose **6**, glucose **7**, mannose **8**, and talose **9** from levoglucosan **1**.

The synthesis of complex polyfluorinated carbohydrates is challenging and new synthetic methods must be developed. Pioneering work by many groups brought significant progress to the development of innovative synthetic methodology to access polyfluorosugars [18]. For our part, we recently described the preparation of multi-vicinal trifluorinated hexopyranose analogues **6–9** using the Chiron approach from levoglucosan (**1**, Figure 1) [19]. Various 1,6-anhydro-2,3-dideoxy-2,3-difluoro-β-ᴅ-hexopyranoses **2–5** were prepared as pivotal intermediates to access compounds **6–9**. We initially supposed that C4 deoxyfluorination of intermediates **2–5** could provide the corresponding trifluorinated product with inversion of configuration. Although that holds true for intermediates **2–4** (leading to products **6–8** after few synthetic steps), intermediate **5** failed to produce any significant results under various reaction conditions. Surprisingly, depending on the reaction conditions, C4 deoxyfluorination of compound **4** led to either the product with inversion or retention of configuration.

Recently, the group of Linclau proposed the involvement of an oxiranium intermediate for the C4 deoxyfluorination of compound **3** using diethylaminosulfur trifluoride (DAST) [20]. Moreover, in their report, they also rationalized our previous finding that a DAST-mediated deoxyfluorination of compound **4** occurs only with retention of configuration via a possible oxiranium intermediate [19]. Hence, we wish to complement this work by proposing an alternative to the DAST reagent for the selective C4 deoxyfluorination of intermediate **4**.

Our group is interested in the synthesis, physical properties and biological activities of heavily fluorinated carbohydrates [19,21–25]. With intermediates **2–5** in hand, we studied the reactivities of a small set of chirally distinct difluorinated 1,6-anhydro-β-ᴅ-hexopyranose analogues. We aimed to explore the difference in reactivities of compounds **2–5** under various deoxyfluorination reaction conditions. In our specific case, we were able to modulate the stereoselectivity of the nucleophilic fluorination at C4 with the use of Et_3_N·3HF. As proposed by the group of Linclau [20], we support the involvement of an oxiranium intermediate (results reinforced by density functional theory (DFT) calculations). Finally, we wish to report the preparation of novel multi-vicinal fluorinated alditol analogues using a simple reduction protocol.

## Results and Discussion

We initially supposed that DAST, a commonly used reagent to install fluorine atoms on the carbohydrate core, would be suitable for the C4 deoxyfluorination of intermediates **2–5**. However, it is well documented that undesired rearrangement products with 1,2-aglycone migration [26–28] or skeletal rearrangements [29] have been observed during fluorodeoxygenation of glycopyranosides with DAST. Nevertheless, we investigated the preparation of 1,6-anhydro-2,3,4-trideoxy-2,3,4-trifluoro-β-ᴅ-hexopyranose analogues from previously described 1,6-anhydro-2,3-dideoxy-2,3-difluoro-β-ᴅ-hexopyranoses **2–5** using DAST (Table 1). We thus used 2 equivalents of DAST at 100 °C under microwave heating for 1 h and reactions were monitored using ^19^F NMR spectroscopy with 2-fluoro-4-nitrotoluene as internal standard. Fluorodeoxygenation of glucopyranose **2** gave galactose **10** (inversion of configuration), as the only diastereoisomer (Table 1, entry 1). Interestingly, the epimer at C4 (compound **3**) provided galactose **10** (retention of configuration) with a 39% yield along with glucose **11** (inversion of configuration) in 31% yield (Table 1, entry 2) [20,30]. Moreover, the use of the talose analogue **4** generated exclusively the product with retention of configuration **12**. This result was surprising since the only difference with **3** is the stereochemistry of the fluorine atom at C2. Finally, only decomposition originated from the use of mannose analogue **5** as substrate. The difference in terms of reactivity between **5** and **2** was unexpected since they only differ from the stereochemistry of the fluorine atom at C2 (distal to the reactive site at C4).

**Table 1:** Synthesis of 1,6-anhydro-2,3,4-trideoxy-2,3,4-trifluoro-β-ᴅ-hexopyranoses **10–12** using DAST.

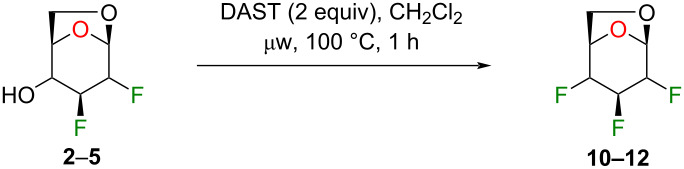			

Entry	Starting materials	Conversion (%)^a^	Products (yield, %)^a^

1	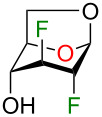 **2**	98	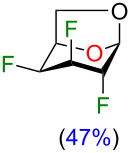 **10**
2	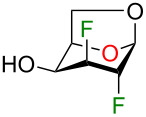 **3**	99	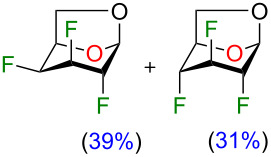 **10** + **11**
3	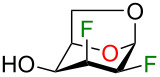 **4**	100	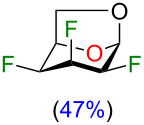 **12**
4	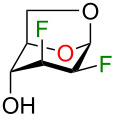 **5**	100	decomposition

^a^Conversions and yields were determined from the ^19^F NMR (470 MHz, CDCl_3_) using 2-fluoro-4-nitrotoluene as internal standard.

**Figure 2:**
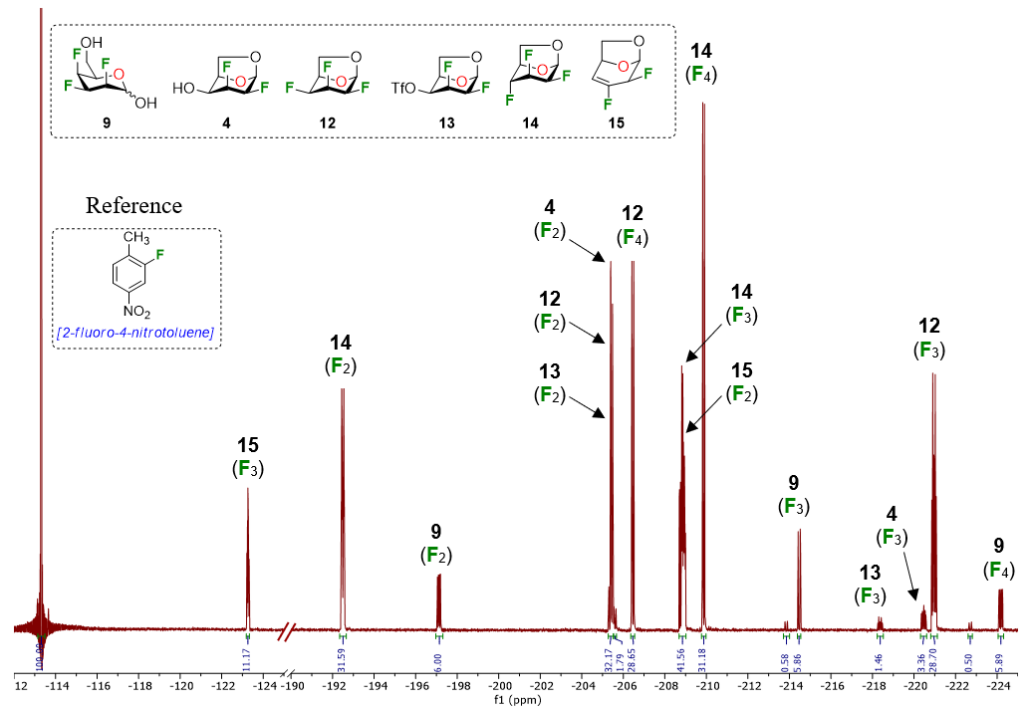
Typical ^19^F NMR spectrum (470 MHz, CDCl_3_) of the crude reaction mixture using Et_3_N·3HF/Et_3_N (entry 3 of Table 2).

Although rather disappointing in terms of yields, these results shed some light on the fact that both the stereochemistry of the fluorine atom at C2 and the C4 hydroxy group could influence the outcome of the fluorodeoxygenation at C4 for 1,6-anhydro-hexopyranose systems in the studied conditions. At this point, it became obvious to us that it would be difficult to selectively prepare 1,6-anhydro-2,3,4-trideoxy-2,3,4-trifluorohexopyranose analogues using DAST-mediated deoxyfluorination. Consequently, we turned our attention towards activation of the C4 hydroxy group as a triflate followed by exposure to a fluorine nucleophile [31]. We selected Et_3_N·3HF as a simple, stable, and versatile nucleophilic reagent since it would be possible to modulate the nucleophilicity/basicity with addition of Et_3_N [32]. Triflate **13** [19] was engaged as a good substrate model since the product arising from an inversion of configuration could generate the desired trifluorinated mannose analogue **14**, inaccessible from the DAST-mediated fluorodeoxygenation (Table 2) [33]. We first used 15 equivalents of Et_3_N·3HF at 80 °C for 24 h with the addition of 50 equivalents of Et_3_N as solvent (Table 2, entry 1). To our delight, mannose analogue **14** was formed as the major product (44% yield), along with 21% of the talose analogue **12** and the elimination product **15** in 20% yield, as determined after analysis of the ^19^F NMR spectra of the crude reaction mixture. Figure 2 shows a representative ^19^F NMR spectrum (470 MHz, CDCl_3_) of the crude reaction mixture of the fluorodeoxygenation using Et_3_N·3HF (entry 3 of Table 2). Next, decreasing the amount of Et_3_N allowed us to generate in situ Et_3_N·1HF (Table 2, entry 2) and Et_3_N·1.5HF (Table 2, entry 3). Using in situ generated Et_3_N·1HF, the mannose analogue **14** was formed in 48% yield and with Et_3_N·1.5HF, compound **14** was formed in 32% yield. It is known that Et_3_N·3HF/Et_3_N is more nucleophilic than Et_3_N·3HF with a limited basicity that reduces the formation of elimination byproducts [32,34]. Then, screening other bases (Table 2, entries 4–6) did not furnish the mannose analogue **14** in higher yield and using pyridine·1HF as reagent (pyridine·9HF with 120 equivalents of pyridine) allowed only poor conversion of starting material. Finally, performing the reaction in neat Et_3_N·3HF (no additive) provided almost exclusively talose analogue **12** with no elimination byproduct. These results suggest that addition of Et_3_N dictates the selectivity for the C4 fluorination.

**Table 2:** Selective synthesis of trifluorotalose analogue **12** or trifluoromannose analogue **14** using Et_3_N·3HF.^a^

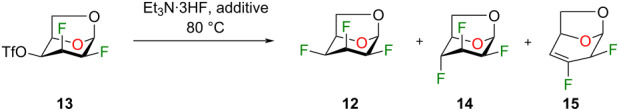					

Entry	Additive (equiv)	Conversion^b^ (%)	Yield (%)^b^		

			**12**	**14**	**15**

1^a^	Et_3_N (50)	100	21	44	20
**2**	**Et_3_N (30)**	**100**	**22**	**48**	**15**
3	Et_3_N (15)	98	29	32	11
4	quinuclidine (30)	100	6	18	22
5	pyridine (30)	92	29	6	4
6	(−)-sparteine (30)	99	10	43	14
7^c^	pyridine (120)	46	1	0	2
**8**	**–**	**98**	**70**	**3**	**0**

^a^Reactions were carried out in a glass seal tube with 15 equivalents of Et_3_N·3HF at 80 °C for 24 h. ^b^Conversions and yields were determined with the ^19^F NMR (470 MHz, CDCl_3_) using 2-fluoro-4-nitrotoluene as internal standard. ^c^Pyridine·9HF was used instead of Et_3_N·3HF.

**Figure 3:**
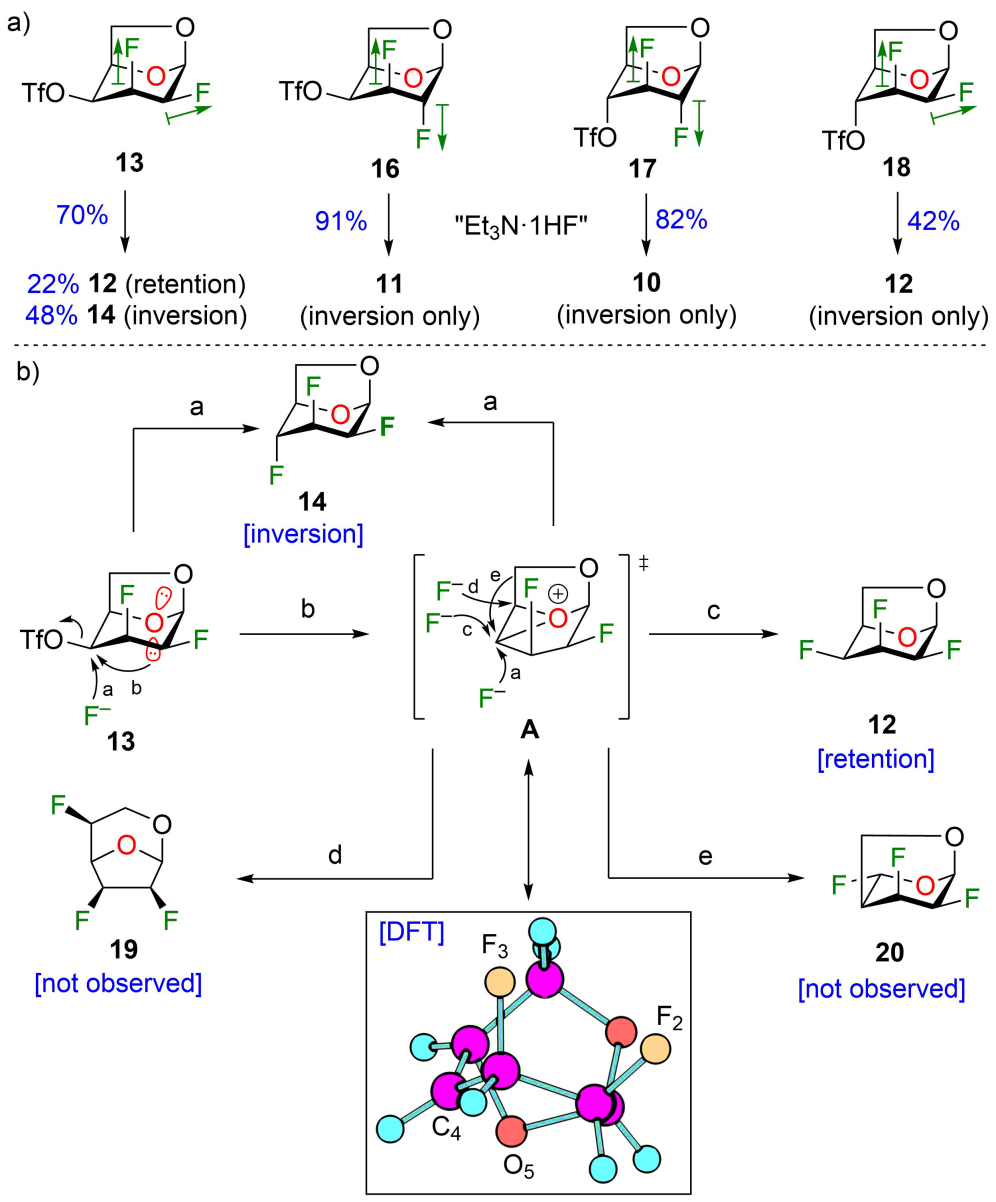
Fluorination at C4 of 1,6-anhydro-2,3-difluorohexopyranose analogues. a) Reactions on triflates **13**, **16–18**, dipole of C–F bonds are displayed in green arrows; b) Proposed mechanism for the formation of trifluorotalose analogue **12** and trifluoromannose analogue **14** via an oxiranium-like intermediate **A** from compound **13**.

With the optimized conditions in hand, we investigated the use of other triflate analogues (Figure 3a). Under optimized conditions (Table 2, entry 2), triflate analogues **16–18** [19] gave exclusively products with inversion of configuration, although in a lower yield for compound **18**. These results highlighted the likely influence of the stereochemistry of the fluorine atom at C2 in particular when we compared the reactivity between compounds **13** and **16**. The nucleophilic substitution with retention of configuration was unexpected since the fluoride anion would have to approach the more sterically hindered β-face. To account for the retention of configuration (minor product) in the fluorination of compound **13**, we proposed the involvement of an oxiranium-like intermediate **A** (Figure 3b). Interestingly, oxiranium ions in carbohydrate chemistry have been proposed as intermediates in the course of various reactions [20,29,35–36]. An equatorial fluorine atom at C2 (antiperiplanar to the C1–O5 bond) reduces the endocyclic oxygen polarizability, but also destabilizes a possible carbocation at C4 (the C2–F2 bond is antiperiplanar to the C3–C4 bond) [37]. As a result, the oxiranium ion could be stabilized in the presence of triethylamine. A lone pair of electrons on the nitrogen atom from triethylamine overlaps with the antibonding σ* orbital of the O5–C4 bond of the oxirane, favoring the fluorine electron density to attack equatorially. Although we propose an oxiranium intermediate for the C4 deoxyfluorination of **13**, we did not observe any ring rearrangement, typically as a result of ring contraction (for example, compound **19**, step d) [26,29,38]. Also, we did not observe any 1,2-alkyl shift of C6 from C5 to C4 with enlargement of the dioxolane ring followed by fluorine introduction at C5 (compound **20**, step e) [33]. This known alkyl shift [29,39] is usually displayed by more carbocationic character of the intermediate species [40]. In our case, the polyfluoroalkyl group could destabilize the adjacent carbocation center [41–43], avoiding 1,2-alkyl shift, and thus formation of byproduct **20**. Similarly, the large dipole moment of the C–F bond at C2 influences the outcome of the deoxyfluorination at C4 [3]. For triflates **16** and **17**, both dipoles (green arrows, Figure 3a) are opposite to each other (dipolar relaxation) and consequently provided higher yields for the nucleophilic fluorination at C4 with inversion of configuration. In contrast, triflates **13** and **18** (both dipole in the same direction) led to lower yields for the fluorination with inversion of configuration. Finally, DFT calculations were performed with Gaussian 09, revision E.01 [44] to evaluate the hypothesis for the formation of oxiranium ion **A**. Calculations were performed with the CAM-B3LYP functional [45–47] using Grimme’s D3 dispersion correction [48] and the 6-31+G(d,p) basis set. The results of our modeling calculations showed a distance of 1.52 Å between the endocyclic oxygen and C4.

**Scheme 1:**
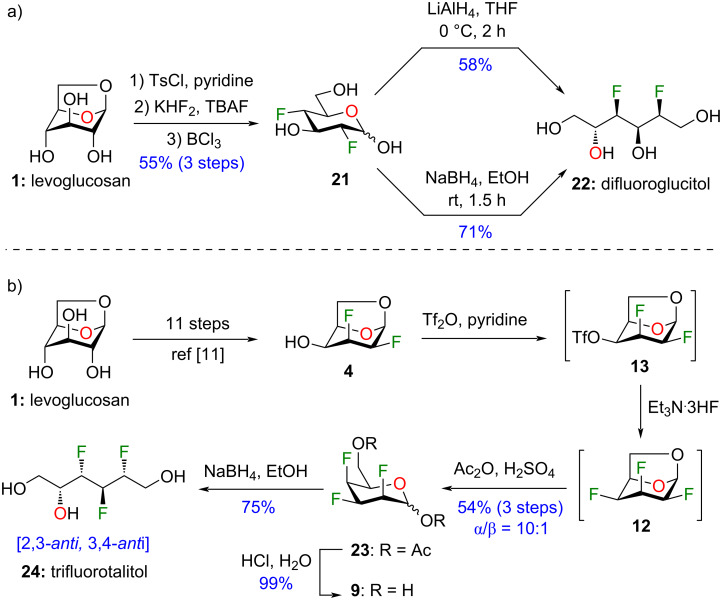
Synthesis of polyfluorinated alditols from levoglucosan **1**: a) difluoroglucitol analogue **22**; b) trifluorotalitol analogue **24**.

With the optimisation of the C4 deoxyfluorination successfully completed, and as a demonstration of the usefulness of our strategy, we completed the synthesis of two fluorinated alditol analogues (Scheme 1). We first evaluated reduction conditions on a simpler difluorinated hexopyranose analogue. Thus, difluoroglucose **21**, easily accessible in 3 steps from levoglucosan (**1**) [21], was subjected to lithium aluminium hydride (LiAlH_4_) in THF (Scheme 1a) and difluoroglucitol **22** was isolated in 58% yield. The reaction was hardly reproducible because a thick gel was formed after neutralisation with an acidic resin, thus resulting in yield loss after a difficult filtration. Next, we evaluated sodium borohydride (NaBH_4_) as reducing reagent. Difluoroglucose **21** was subjected to 5 equivalents of NaBH_4_ in EtOH at rt and compound **22** was isolated in 71% yield. We then apply this strategy to the synthesis of trifluorohexitol analogue integrating fluorine atoms at C2, C3, and C4 (Scheme 1b). Compound **4**, also accessible from levoglucosan (**1**) [19], was activated as triflate and subjected to nucleophilic fluorination. The crude reaction mixture was treated under acidic conditions and furnished the desired acetylated analogue **23** in 54% over 3 steps. This synthetic sequence represents a useful alternative to DAST or TBAF (tetrabutylammonium fluoride) that lead to elimination byproducts. Finally, after acetates removal, reduction of compound **9** with sodium borohydride led to novel trifluorotalitol analogue **24** in 75% yield containing a 2,3*-anti*, 3,4*-anti* pattern for the integrated fluorine atoms.

## Conclusion

Supporting evidence from numerous studies on various substrates indicated that predicting the selectivity of the DAST-mediated deoxyfluorination is a difficult task to achieve. The simple synthetic approach described herein and the use of Et_3_N·3HF as an alternative to the unpredictable DAST looked promising. In the specific case of triflate **13**, we were able to modulate the stereoselectivity of the nucleophilic fluorination at C4 using simple Et_3_N·3HF with or without addition of triethylamine. The role of the reagent and the remote influence of the fluorine atom at C2 on reactivity at C4 could concomitantly contribute to a new fluorine effect in nucleophilic substitution [49]. Finally, the synthesis of two distinct polyfluorinated alditol analogues was achieved. The strategy described herein will allowed us to achieve the synthesis of other polyfluorinated hexitol analogues in order to explore their physical properties.

## Supporting Information

File 1 Detailed experimental procedures, characterization data, copies of ^1^H, ^13^C, ^19^F, COSY, and HSQC NMR spectra of all new compounds, and optimization of the deoxyfluorination.
