# Supplementary material for: Fluorine effect in nucleophilic fluorination at C4 of 1,6-anhydro-2,3-dideoxy-2,3-difluoro-β-D-hexopyranose
Source: Beilstein J Org Chem. 2020 Nov 25;16:2880–7. doi: 10.3762/bjoc.16.237 (PMC7705882; doi:10.3762/bjoc.16.237)
Supplement: File 1 — Detailed experimental procedures, characterization data, copies of 1H, 13C, 19F, COSY, and HSQC NMR spectra of all new compounds, and optimization of the deoxyfluorination. [file Beilstein_J_Org_Chem-16-2880-s001.pdf]

BEILSTEIN JOURNAL OF ORGANIC CHEMISTRY

Supporting Information

for

Fluorine effect in nucleophilic fluorination at C4 of 1,6-anhydro-2,3-dideoxy-2,3-difluoro-β-D-hexopyranose

Danny Lainé, Vincent Denavit, Olivier Lessard, Laurie Carrier, Charles-Émile Fecteau, Paul A. Johnson and Denis Giguère

Beilstein J. Org. Chem. **2020,** *16,* 2880–2887. doi:10.3762/bjoc.16.237

Detailed experimental procedures, characterization data, copies of ^{1}H, ^{13}C, ^{19}F, COSY, and HSQC NMR spectra of all new compounds, and optimization of the deoxyfluorination

License and Terms: This is a supporting information file under the terms of the Creative Commons Attribution License (https://creativecommons.org/licenses/by/4.0). Please note that the reuse, redistribution and reproduction in particular requires that the authors and source are credited.
The license is subject to the *Beilstein Journal of Organic Chemistry* terms and conditions: (https://www.beilstein-journals.org/bjoc)

I. Experimental section

General methods **S2**

Optimization of the fluorodeoxygenation using $Et_3N{\cdot}3HF$ **S3**

General procedures **S5**

II. Density functional theory calculations **S8**

III. NMR spectra of compounds **S9**

IV. References **S30**

I. Experimental section

General methods

All reactions were carried out under an argon atmosphere with dry solvents under anhydrous conditions, unless otherwise noted. Dry dichloromethane (CH_2Cl_2) was obtained by passing commercially available pre-dried, oxygen-free formulations through activated alumina columns using a Vacuum Atmospheres Inc. Solvent Purification System. Yields refer to chromatographically and spectroscopically (^{1}H NMR) homogeneous materials, unless otherwise stated. Reagents were purchased at the highest commercial quality available and used without further purification, unless otherwise stated. Reactions were monitored by thin-layer chromatography (TLC) carried out on 0.25 mm E. Merck silica gel plates (60F-254) using UV light as visualizing agent and charring with 1.5 g of $KMnO_4$, 10 g K_2CO_3, and 1.25 mL 10% NaOH in 200 mL of water, followed by heating with a heatgun as developing agents. SiliaFlash® P60 (particle size 40–63 mm, 230– 400 mesh) was used for flash column chromatography. NMR spectra were recorded on an Agilent DD2 spectrometer (at 500 MHz for ^{1}H, 470 MHz for ^{19}F, and 126 MHz for ^{13}C) and calibrated using residual undeuterated solvent peaks ($CDCl_3$ ^{1}H δ = 7.26 ppm, ^{13}C δ = 77.16 ppm; acetone-d_6: ^{1}H δ = 2.05 ppm, ^{13}C δ = 29.84 ppm) as an internal reference. ^{19}F NMR spectra were calibrated using hexafluorobenzene, which gives a signal at ^{19}F δ = −162.29 ppm with respect to that of the reference compound $CFCl_3$. Coupling constants (*J*) are reported in Hertz (Hz), and the following abbreviations were used to designate multiplicities: s = singlet, d = doublet, t = triplet, q= quartet, p = quintet, m = multiplet, br = broad. Assignments of NMR signals were made by homonuclear (COSY) and heteronuclear (HSQC, HMBC, and ^{19}F gc2HSQC) two-dimensional correlation spectroscopy. Infrared (IR) spectra were recorded using an ABB Bomem MB-Series Arid Zone FTIR MB-155 Spectrometer, with a ZnSe crystal plate. The absorptions are given in wavenumbers (cm^{-1}). High resolution mass spectra (HRMS) were measured with an Agilent 6210 LC Time of Flight mass spectrometer in electrospray mode (ESI). Either ammonium adducts $[M + NH_4]^+$ or deprotonated molecular ions $[M - nH]^{n-}$ were used for empirical formula confirmation. Optical rotations were recorded on a JASCO DIP-360 digital polarimeter at 589 nm and are reported in units of 10^{-1} (deg cm^2 g^{-1}).

Optimization of the fluorodeoxygenation using $Et_3N{\cdot}3HF$

Table S1. Optimization of the selective synthesis of trifluorotalose analogue **12** and trifluoromannose analogue **14** using $Et_3N{\cdot}3HF$.

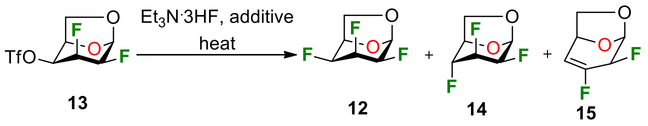

Entry[a]	Additive (equiv)	Temperature (°C)	Conversion (%)[b]	Yields (%)[b]		
				**12**	**14**	**15**
1	Et_3N (50)	80	100	21	44	20
2	DIPEA (50)	80	98	23	36	15
3[c]	Et_3N (50)	80	77	21	11	4
4[c]	Et_3N (50)	80	87	25	12	3
5[d]	Et_3N (50)	80	30	6	15	7
6[d]	Et_3N (50)	90	50	12	24	10
7[d]	Et_3N (50)	100	99	29	38	22
8	Et_3N (15)	80	98	29	32	11
9	Et_3N (23)	80	97	33	34	12
**10**	**Et_3N (30)**	**80**	**100**	**22**	**48**	**15**
11	Et_3N (30)	70	79	7	19	7
12	Et_3N (30)	90	100	14	23	11
13	Et_3N (30)	100	100	18	30	19
14[e]	Et_3N (30)	80	96	19	45	20
15[f]	Et_3N (30)	80	100	24	48	21
16	quinuclidine (30)	80	100	6	18	22
17	pyridine (30)	80	92	29	6	4
18	(−)-sparteine (30)	80	99	10	43	14
19	DBU (30)	80	100	0	25	67
20[g]	pyridine (120)	80	46	1	0	2
**21**	–	**80**	**98**	**70**	**3**	**0**

[a]Reactions were carried out in a glass seal tube with 15 equivalents of $Et_3N{\cdot}3HF$ for 24 h unless otherwise stated.
[b]Conversions and yields were determined with the ^{19}F NMR (470 MHz, $CDCl_3$) using 2-fluoro-4-nitrotoluene as internal standard.
[c]Reactions were carried out in a PTFE test tube instead of a glass seal tube for 24 h (entry 3), and 48 h (entry 4).
[d]Reactions were heated in a microwave reactor for 2 h.
[e]The reaction was stirred for 12 h.
[f]The reaction was stirred for 48 h.
[g]Pyridine·9HF was used instead of $Et_3N{\cdot}3HF$.

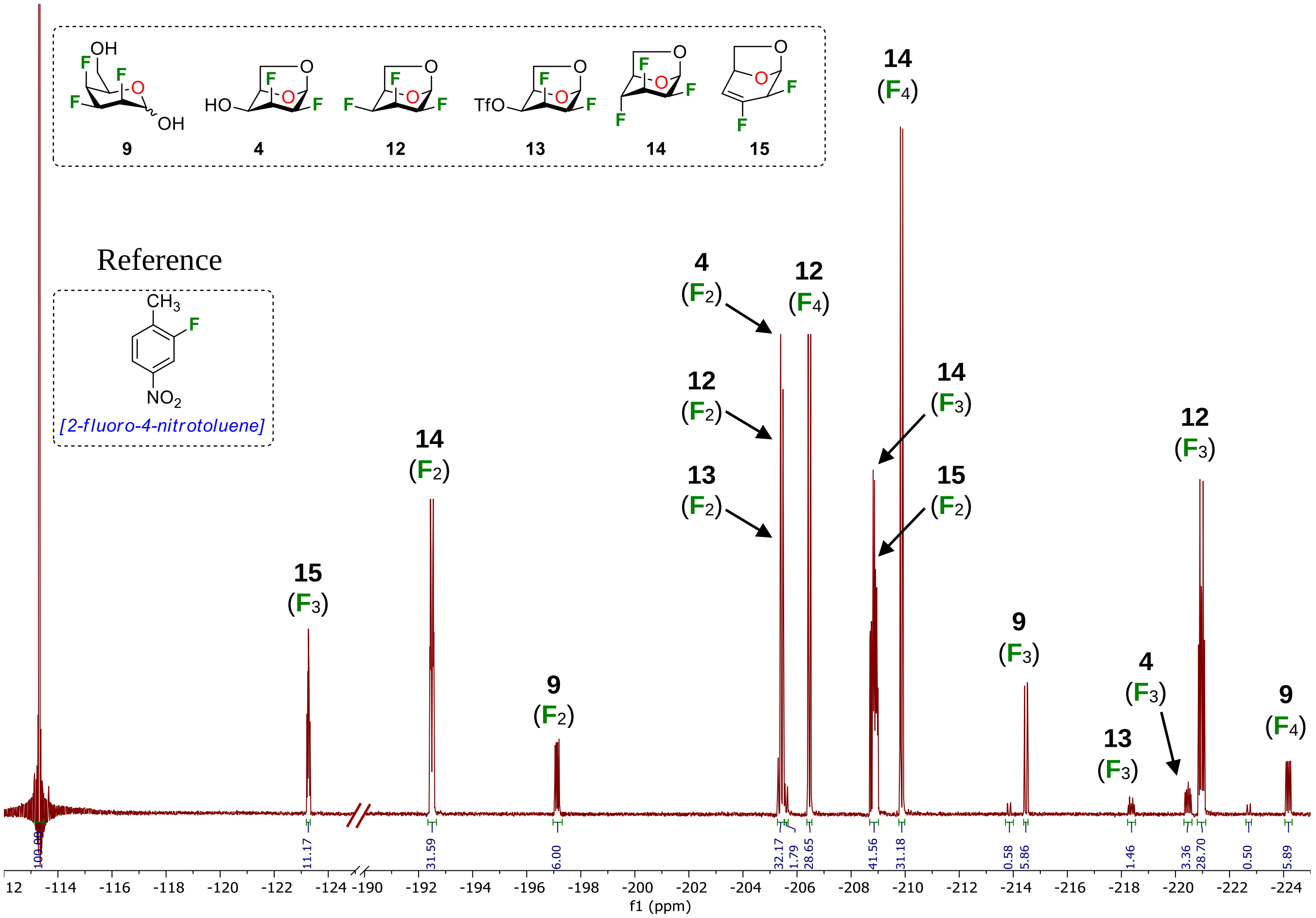

Figure S1. Typical ^{19}F NMR spectrum (470 MHz, $CDCl_3$) observed for the fluorodeoxygenation using $Et_3N{\cdot}3HF$ (entry 8 of Table S1).

General Procedures

General procedure I: C4 deoxyfluorination conditions using DAST (Table 1)

To a stirred solution of the starting 1,6-anhydro-2,3-dideoxy-2,3-difluoro-β-D-hexopyranoses **2–5** (1 equiv) in CH_2Cl_2 (0.1 M) was added DAST (1 equiv) and 2-fluoro-4-nitrotoluene (1 equiv). The resulting mixture was heated in a microwave reactor at 100 °C for 1 h. After this time, $CDCl_3$ (0.5 mL was added, and a ^{19}F NMR spectrum of the resulting mixture was taken to give an NMR yield of products **10–12**.

2,4-Dideoxy-2,4-difluoroglucitol (**22**). Reduction using $LiAlH_4$: To a stirred solution of difluoroglucose **21**[1] (51.0 mg, 0.1644 mmol, 1.0 equiv) in THF (1.6 mL) at 0 °C, was added 1 M $LiAlH_4$ in THF (0.67 mL, 0.6740 mmol, 5.0 equiv). The resulting mixture was stirred at 0 °C for 2 h then 2 mL of MeOH was added, and the mixture was neutralized to pH ≈ 7 with acidic resin (Amberlite IR-120). The mixture was filtered and concentrated under reduced pressure. The obtained crude product was purified by flash column chromatography (silica gel, MeOH/CH_2Cl_2 1:9) to give **22** as a white amorphous solid (17.8 mg, 0.0954 mmol, 58% yield). Reduction using $NaBH_4$: To a stirred solution of difluoroglucose **21** (15.4 mg, 0.0836 mmol, 1.0 equiv) in ethanol (1.0 mL) at room temperature was added $NaBH_4$ (16.0 mg, 0.4182 mmol, 5.0 equiv). The resulting mixture was stirred at room temperature for 1.5 h then 1 mL of methanol was added, and the mixture was neutralized to pH ≈ 7 with acidic resin (Amberlite IR-120). The mixture was filtered and concentrated under reduced pressure. The obtained crude product was purified by flash column chromatography (silica gel, MeOH/CH_2Cl_2 1:9) to give **22** as a white amorphous solid (11.0 mg, 0.0591 mmol, 71% yield): R_f = 0.18 (silica, MeOH/CH_2Cl_2 1:9); $[\alpha]_D^{25}$ = −7.08 (c 0.5, MeOH); IR (ATR, diamond crystal) ν 3308, 2935, 2841, 1425, 1231, 1032, 865 cm^{-1}; ^1H NMR (500 MHz, Acetone-d_6) δ 4.63 (dtd, J = 48.3, 5.6, 3.3 Hz, 1H, H2), 4.59 (ddd, J = 46.8, 7.9, 2.0 Hz, 1H, H4), 4.37 (d, J = 6.5 Hz, 1H, O*H*3), 4.20 (ddtd, J = 29.6, 17.3, 6.0, 1.9 Hz, 1H, H3), 4.21 – 4.14 (m, 1H, O*H*5), 4.10 (t, J = 5.8 Hz, 1H, O*H*1), 4.01 – 3.96 (m, 1H, H5), 3.94 – 3.71 (m, 4H, O*H*6, H1a, H1b, H6a), 3.67 – 3.60 (m, 1H, H6b) ppm; ^{13}C {^1H} NMR (126 MHz, Acetone-d_6) δ 96.1 (dd, J = 173.4, 3.3 Hz, 1C, C2), 92.4 (dd, J = 175.7, 5.5 Hz, 1C, C4), 70.3 (d, J = 25.4 Hz, 1C, C5), 69.3 (dd, J = 21.1, 18.0 Hz, 1C, C3), 63.4 (d, J = 3.8 Hz, 1C, C6), 62.1 (dd, J = 22.7, 2.0 Hz, 1C, C1) ppm; ^{19}F NMR (470 MHz, Acetone-

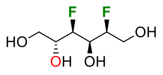

22

d_6) δ -200.01 (dtdd, J = 48.3, 25.2, 17.1, 2.9 Hz, 1F, F2), -209.38 (ddd, J = 46.5, 29.5, 7.3 Hz, 1F, F4) ppm; HRMS calcd for $C_6H_{16}O_4NF_2^+$ $[M + NH_4]^+$ 204.1042 found 204.1046.

1,6-Di-*O*-acetyl-2,3,4-trideoxy-2,3,4-trifluoro-α/β-D-talopyranose (**23**). This compound was prepared

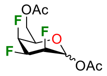

23

in a similar manner as described previously.[2] To a stirred solution of difluorotalose **4**[3] (142.9 mg, 0.8602 mmol, 1.0 equiv) in CH_2Cl_2 (8.6 mL) at room temperature was added pyridine (0.21 mL, 2.580 mmol, 3.0 equiv) and 1 M Tf_2O solution in CH_2Cl_2 (1.3 mL, 1.290 mmol, 1.5 equiv). The resulting mixture was stirred for 1 h then quenched with a saturated aqueous $NaHCO_3$ solution (10 mL). The mixture was extracted with CH_2Cl_2 (3 × 15 mL), and the combined organic phases were washed with a saturated aqueous NaCl solution (40 mL). The organic solution was dried over $MgSO_4$, filtered and concentrated under reduced pressure. The crude triflate **13** was used in the next step without further purification. Following the optimized condition for retention of configuration (Table S1, entry 21), fluorodeoxygenation of triflate **13** was carried with $Et_3N{\cdot}3HF$ (2.1 mL, 12.903 mmol, 15.0 equiv) allowing formation of intermediate **12**. After 24 h, the mixture was cooled down to 0 °C, Ac_2O (16 mL, 172.0 mmol, 200 equiv) and H_2SO_4 (3.7 mL, 68.82 mmol, 80 equiv) were added. The resulting mixture was stirred at room temperature for 16 h then a saturated aqueous $NaHCO_3$ solution (20 mL) was added and the mixture was stirred for 0.5 h. The mixture was extracted with CH_2Cl_2 (3 × 25 mL) and the combined organic phases were washed with a saturated aqueous $NaHCO_3$ solution (2 × 50 mL), an aqueous 1 M HCl solution (50 mL) and a saturated aqueous NaCl solution (50 mL). The organic solution was dried over $MgSO_4$, filtered, and concentrated under reduced pressure. The obtained crude was purified by flash column chromatography (silica gel, EtOAc/hexanes 2:3) to give an anomeric mixture (α/β, 23:1) of **23** as a colourless thick oil (125.5 mg, 0.4645 mmol, 54% yield). The spectroscopic data derived of **23** match those reported in the literature.[3]

2,3,4-Trideoxy-2,3,4-trifluoro-α/β-D-talopyranose (**9**). This compound was prepared in a similar

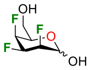

9

manner as described previously.[2] To a stirred solution of compound **23** (47.6 mg, 0.1762 mmol, 1.0 equiv) in water (1.7 mL) at room temperature, was added an aqueous 12 M HCl solution (3.5 mL). The mixture was stirred room temperature for 1 h and then evaporated with a gentle air flow. The obtained yellow crude was purified by flash column chromatography (silica gel, EtOAc/hexanes, 4:1) to give pure product **9** (α/β, 10:1) as a colorless thick oil (32.4 mg, 0.1741 mmol, 99% yield). The spectroscopic data derived of **9** match those reported in the literature.[3]

2,3,4-Trideoxy-2,3,4-trifluorotalitol (**24**). To a stirred solution of trifluorotalose **9** (24.3 mg, 0.1306 mmol, 1.0 equiv) in ethanol (1.3 mL) at room temperature was added $NaBH_4$ (24.7 mg, 0.6528 mmol, 5.0 equiv). The resulting mixture was stirred at room temperature for 1.5 h then 1 mL of methanol was added, and the mixture was neutralized to pH ≈ 7 with acidic resin (Amberlite IR-120). The mixture was filtered and concentrated under reduced pressure. The obtained crude was purified by flash column chromatography (silica gel, MeOH/CH_2Cl_2 1:9) to give **24** as a colorless oil (17.8 mg, 0.0980 mmol, 75% yield): R_f = 0.44 (silica, MeOH/CH_2Cl_2 1:9); $[\alpha]_D^{25}$ = −11.8 (c 0.3, MeOH); IR (ATR, diamond crystal) ν 3312, 2947, 2846, 1420, 1246, 1013, 891 cm^{-1}; ^1H NMR (500 MHz, Acetone-d_6) δ 5.09 (ddddd, *J* = 45.5, 18.6, 10.0, 7.5, 2.6 Hz, 1H, H3), 4.94 (dddddd, *J* = 47.9, 22.5, 6.0, 4.8, 2.6, 1.0 Hz, 1H, H2), 4.92 (dddd, *J* = 46.3, 8.1, 7.6, 2.0 Hz, 1H, H4), 4.32 (br s, 1H, O*H*5), 4.23 (t, *J* = 6.0 Hz, 1H, O*H*1), 4.01 (br s, 1H, O*H*6), 3.92 – 3.80 (m, 3H, H1a, H1b, H5), 3.67 (d, *J* = 6.7 Hz, 2H, H6a, H6b) ppm; ^{13}C {^1H} NMR (126 MHz, Acetone-d_6) δ 94.1 (dd, *J* = 174.3, 21.6, 1.2 Hz, C2), 89.8 (ddd, *J* = 175.6, 27.9, 6.9 Hz, C4), 89.1 (ddd, *J* = 173.3, 28.4, 23.1 Hz, C3), 70.3 (dd, *J* = 18.3, 3.4 Hz, C5), 62.6 (d, *J* = 6.2 Hz, C6), 60.7 (ddd, *J* = 23.5, 8.9, 5.1 Hz, C1) ppm; ^{19}F NMR (470 MHz, Acetone-d_6) δ -200.41 (dddddd, *J* = 47.9, 26.0, 21.0, 18.6, 11.0, 1.5 Hz, 1F, F2), -205.41 (dddddd, *J* = 45.5, 22.5, 13.0, 11.0, 8.1, 2.6 Hz, 1F, F3), -216.67 (dddd, *J* = 46.3, 28.0, 13.0, 10.0 Hz, 1F, F4) ppm; HRMS calcd for $C_6H_{10}O_3F_3^-$ $[M - H]^-$ 187.0588 found 187.0597.

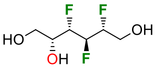

24

II. Density functional theory calculations

Table S2. Optimized cartesian coordinates of oxiranium cation intermediate **A** (CAM-B3LYP-D3/6-31+G(d,p)).

C	-0.899492000	1.097229000	-0.261771000
C	0.562676000	1.346740000	-0.577851000
C	1.596210000	0.638709000	0.186523000
O	1.163799000	0.062757000	-1.130289000
C	0.219976000	-1.094054000	-0.701890000
C	-1.140959000	-0.370839000	-0.686916000
H	-1.557904000	1.792612000	-0.784327000
H	2.617312000	1.004085000	0.133066000
H	0.329312000	-1.863953000	-1.464971000
H	-1.560094000	-0.392002000	-1.698373000
C	1.231873000	-0.404263000	1.244342000
O	0.729129000	-1.507618000	0.475525000
F	-1.107692000	1.257960000	1.087185000
F	-1.986656000	-0.997443000	0.170341000
H	0.487647000	-0.035669000	1.949906000
H	2.116537000	-0.761436000	1.768973000
H	0.851187000	2.189459000	-1.198514000

III. NMR spectra of compounds

^{19}F NMR spectra of the crude mixture reaction described in Table 1 (Entry 1)

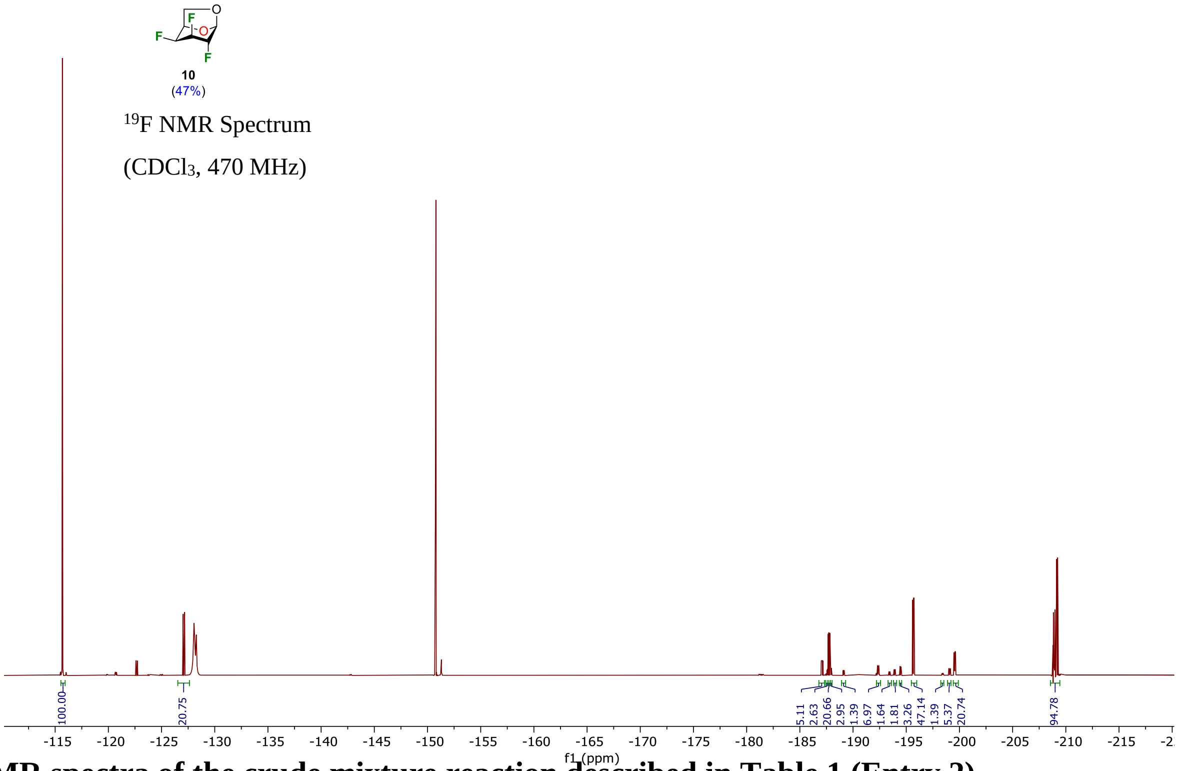

^{19}F NMR spectra of the crude mixture reaction described in Table 1 (Entry 2)

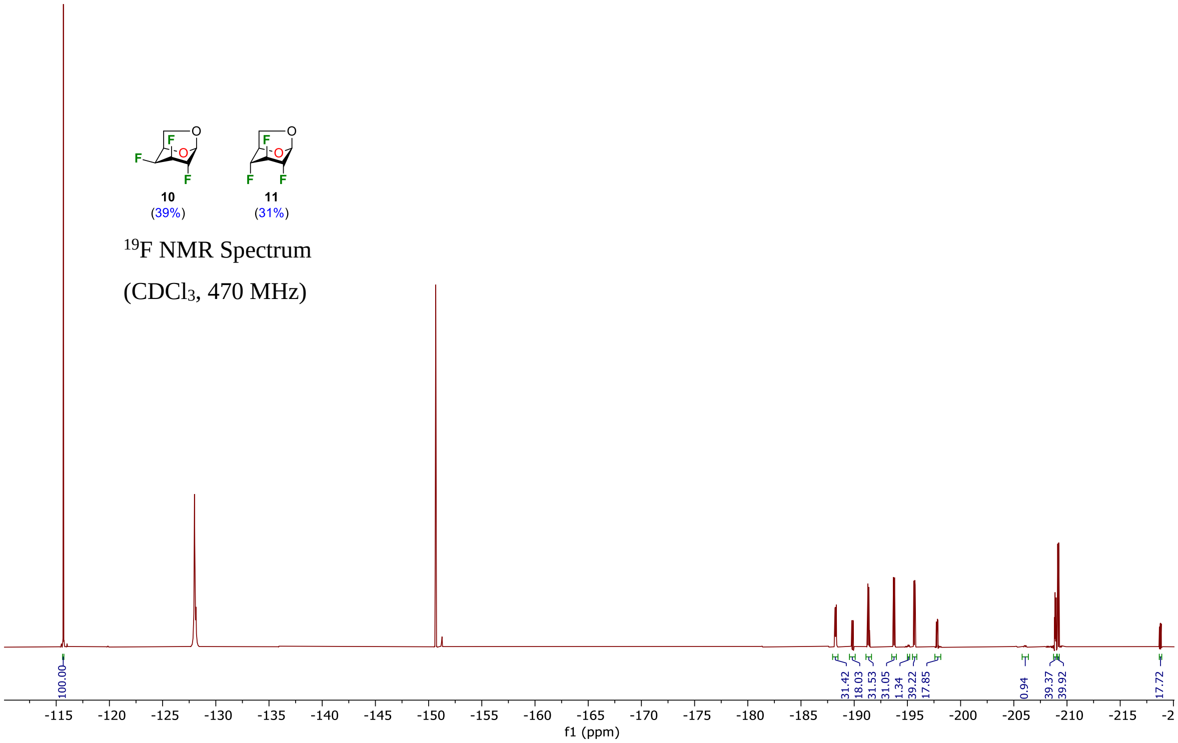

^{19}F NMR spectra of the crude mixture reaction described in Table 1 (Entry 3)

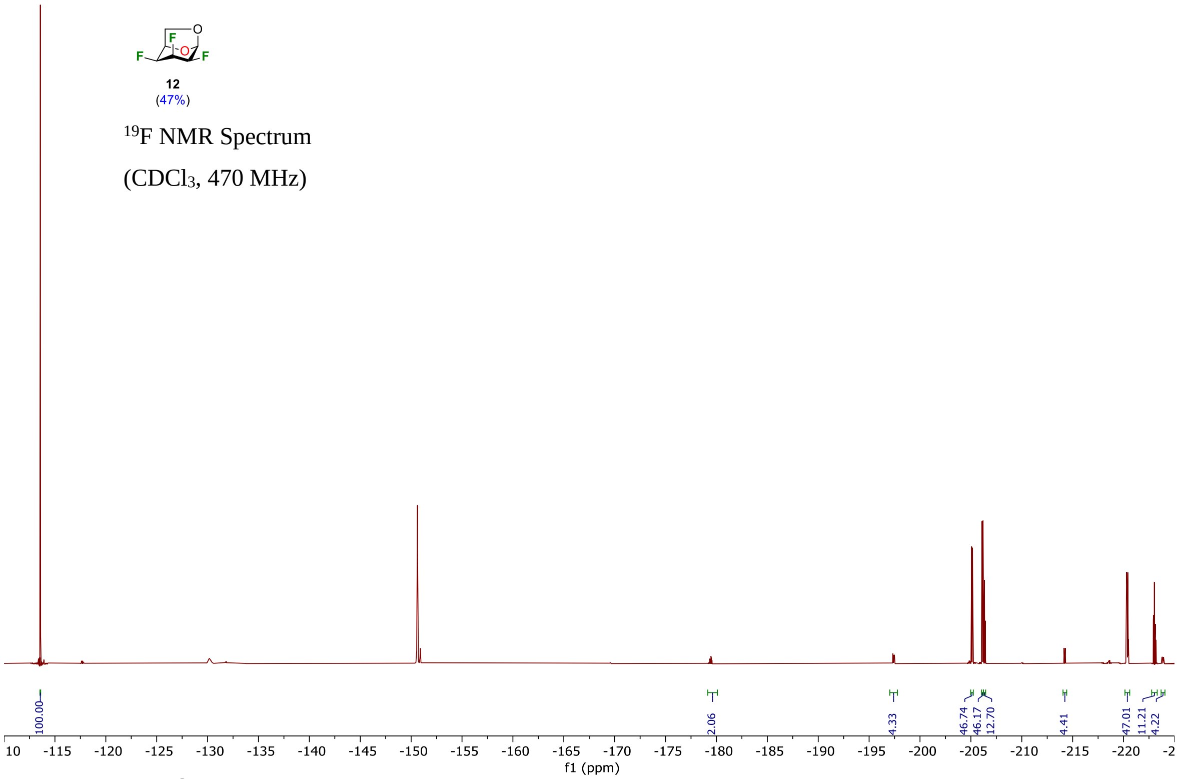

^{19}F NMR spectra of the crude mixture reaction described in Table 1 (Entry 4)

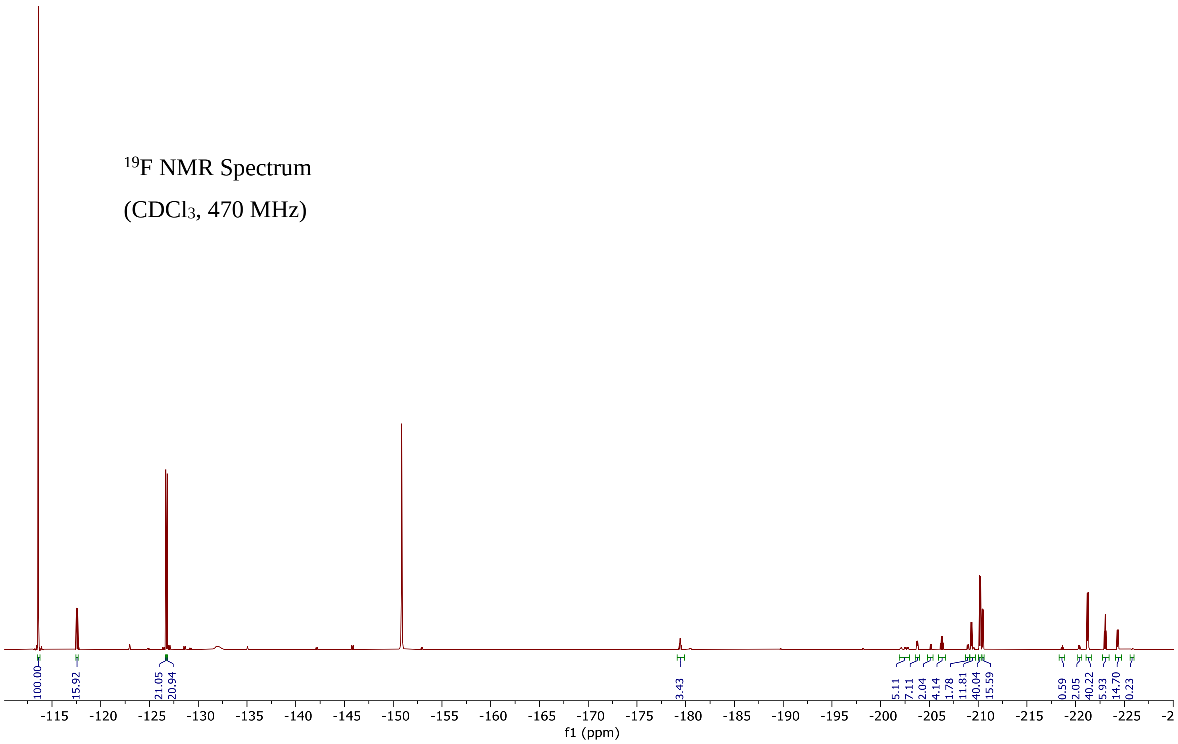

Compound 2

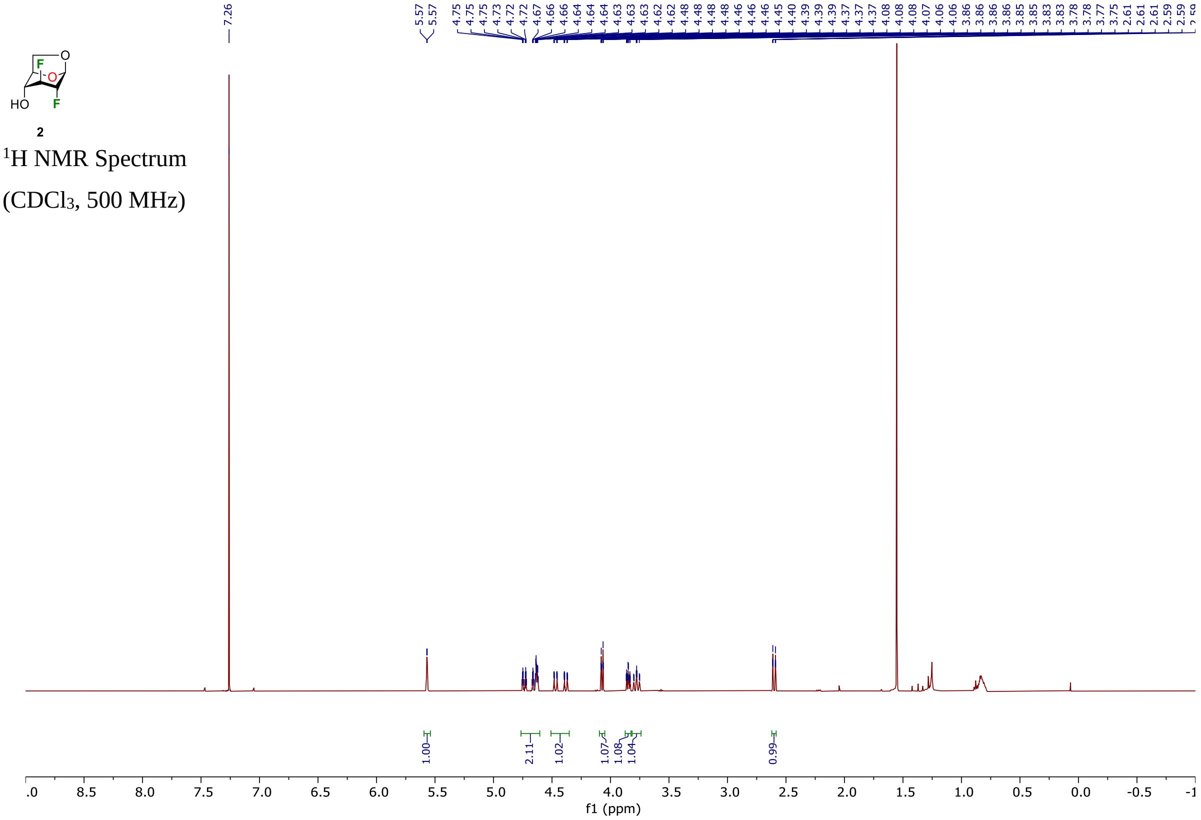

2

^{1}H NMR Spectrum

($CDCl_3$, 500 MHz)

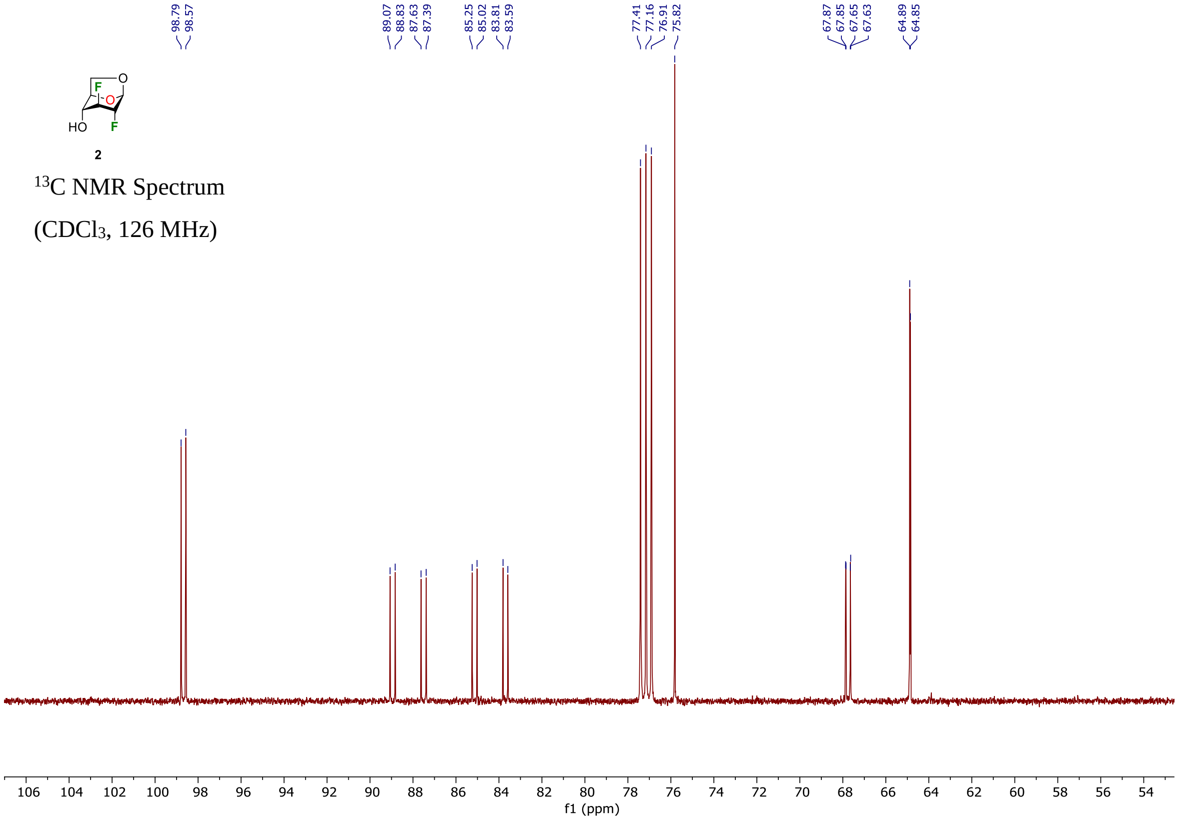

2

^{13}C NMR Spectrum

($CDCl_3$, 126 MHz)

2

^{19}F NMR Spectrum

($CDCl_3$, 470 MHz)

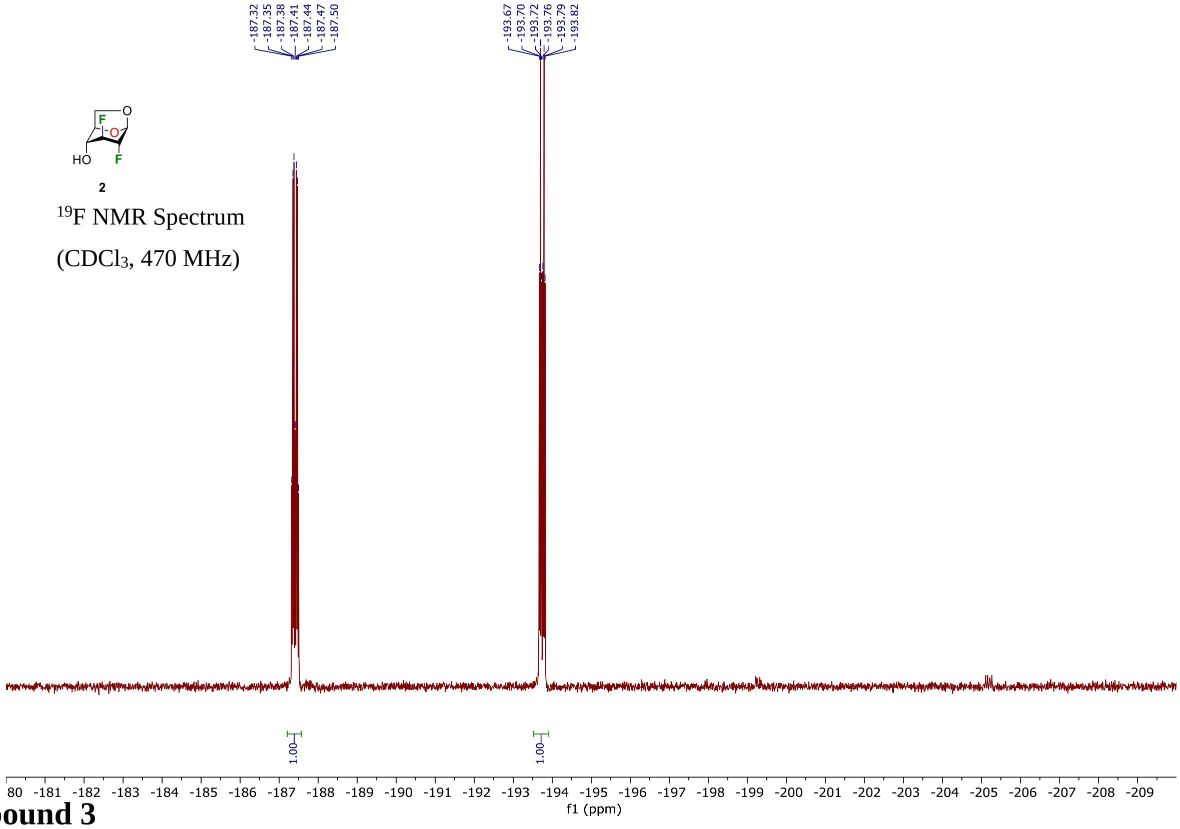

Compound 3

3

^{1}H NMR Spectrum

($CDCl_3$, 500 MHz)

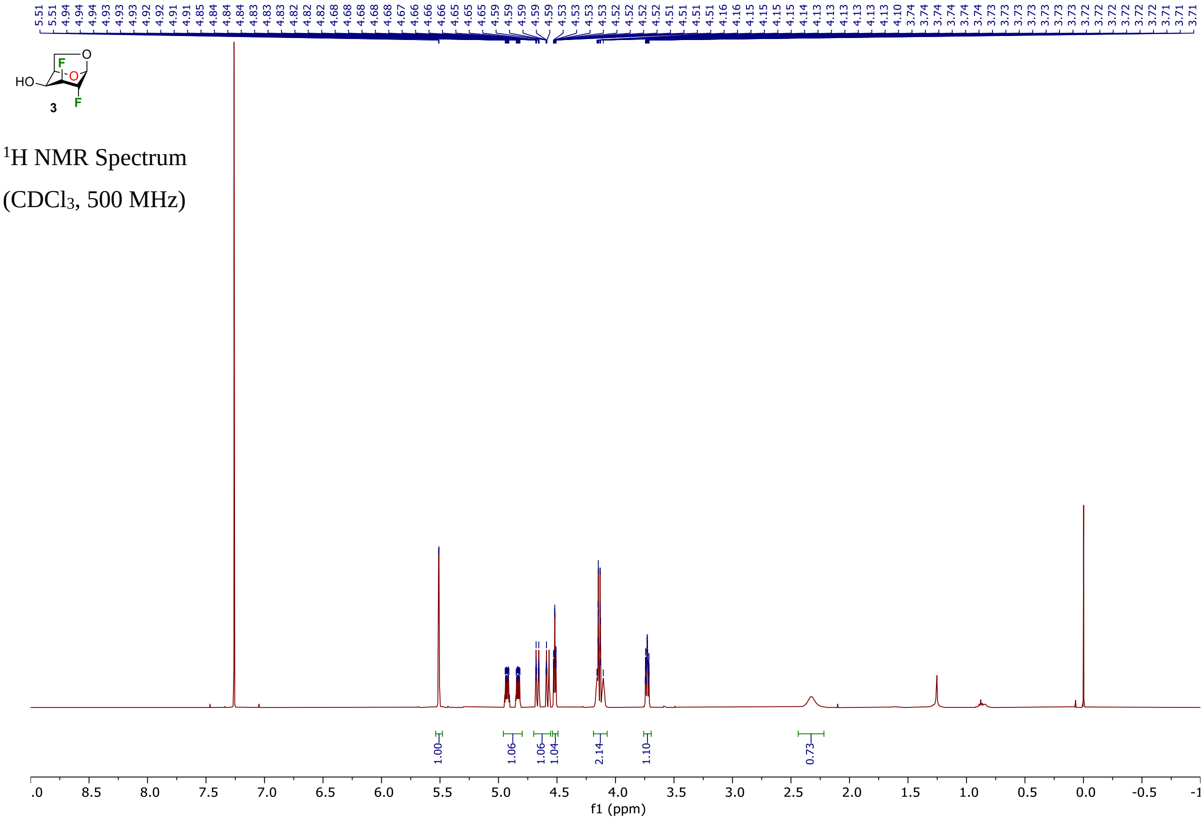

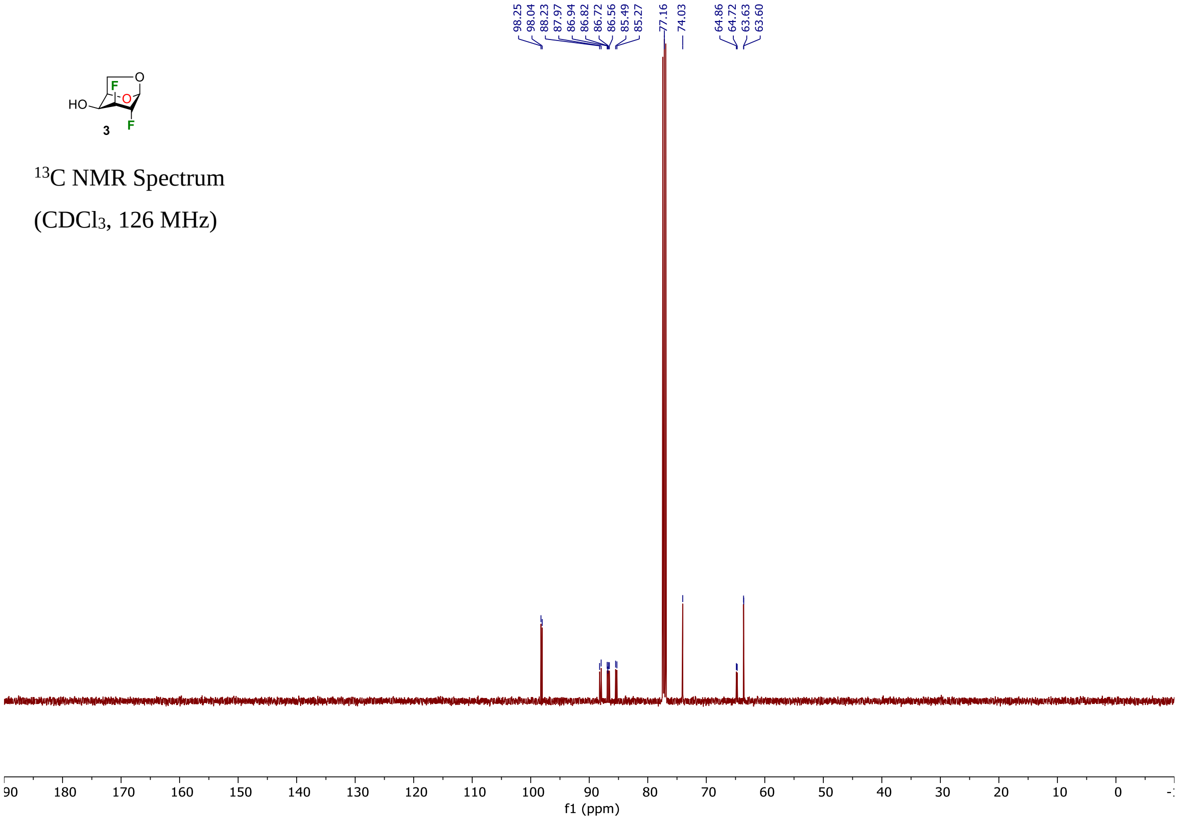

3

^{13}C NMR Spectrum

($CDCl_3$, 126 MHz)

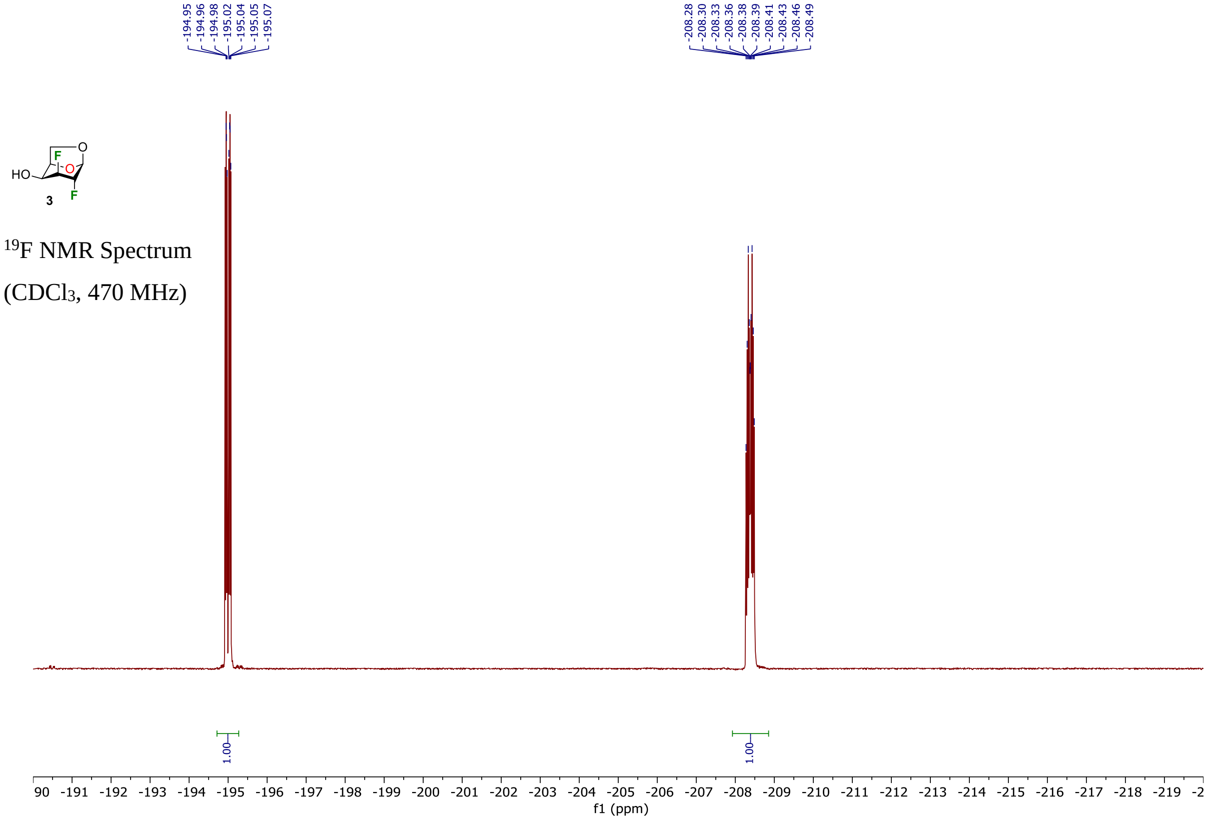

3

^{19}F NMR Spectrum

($CDCl_3$, 470 MHz)

Compound 4

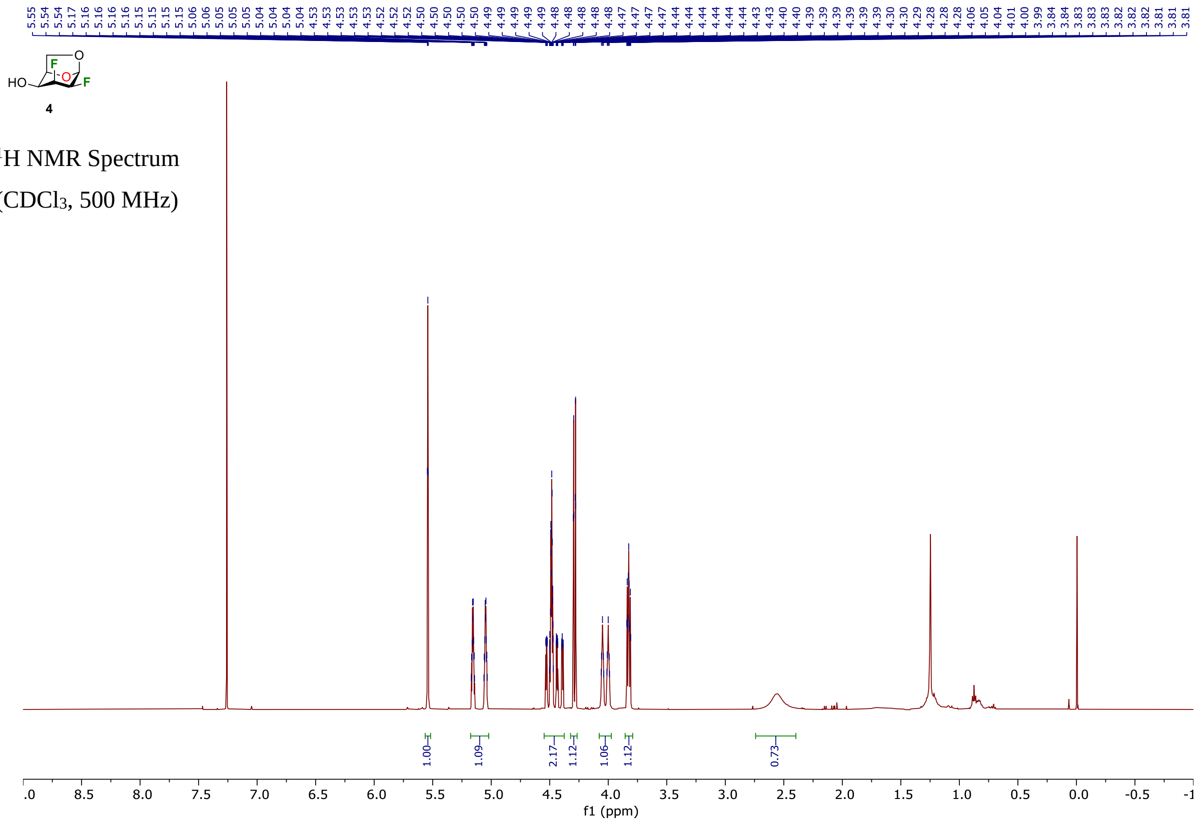

^{1}H NMR Spectrum
(CDCl$_3$, 500 MHz)

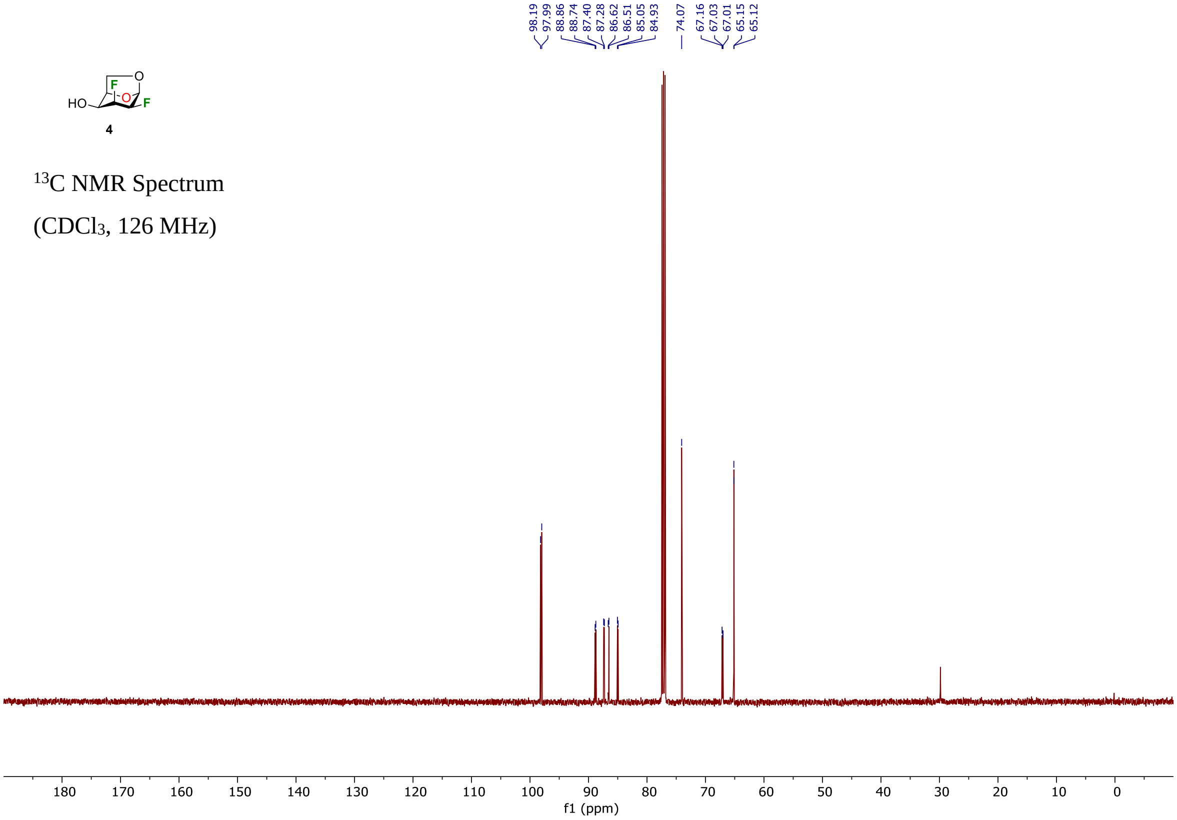

^{13}C NMR Spectrum
(CDCl$_3$, 126 MHz)

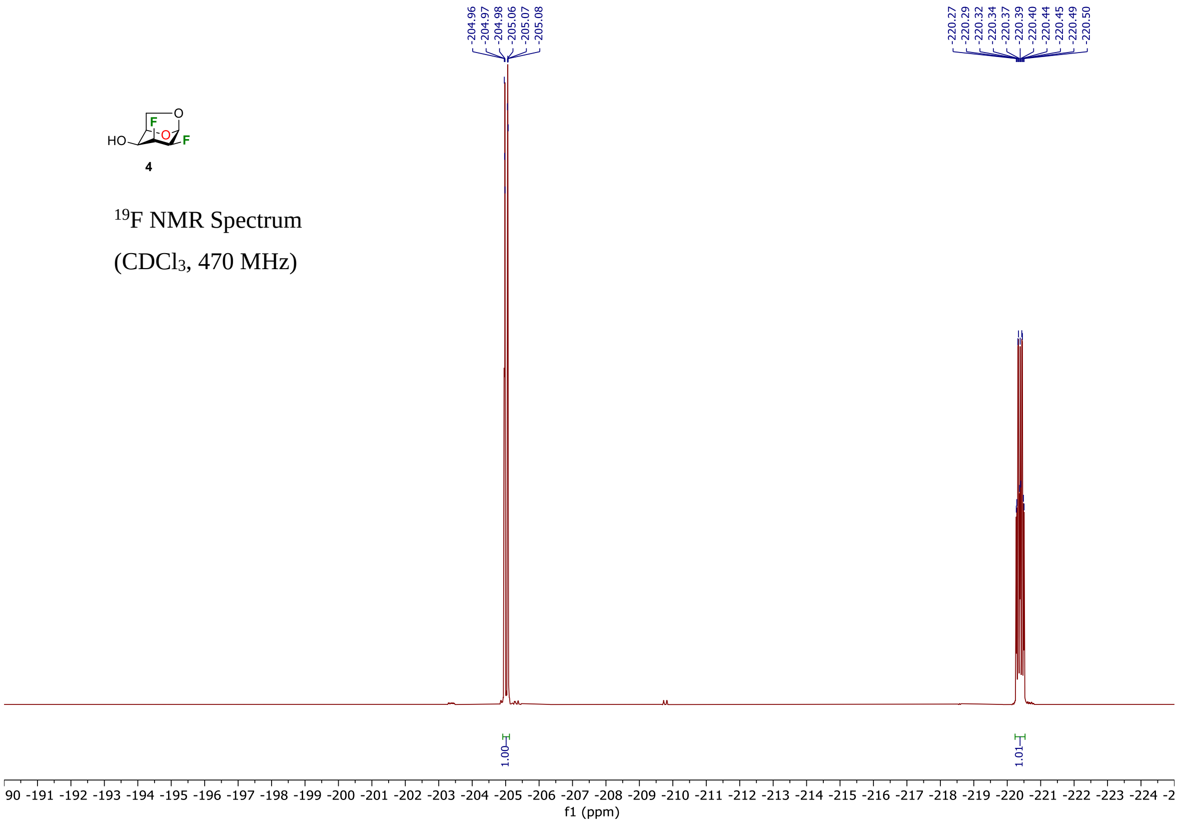
4
^{19}F NMR Spectrum
(CDCl$_3$, 470 MHz)
f1 (ppm)

Compound 5

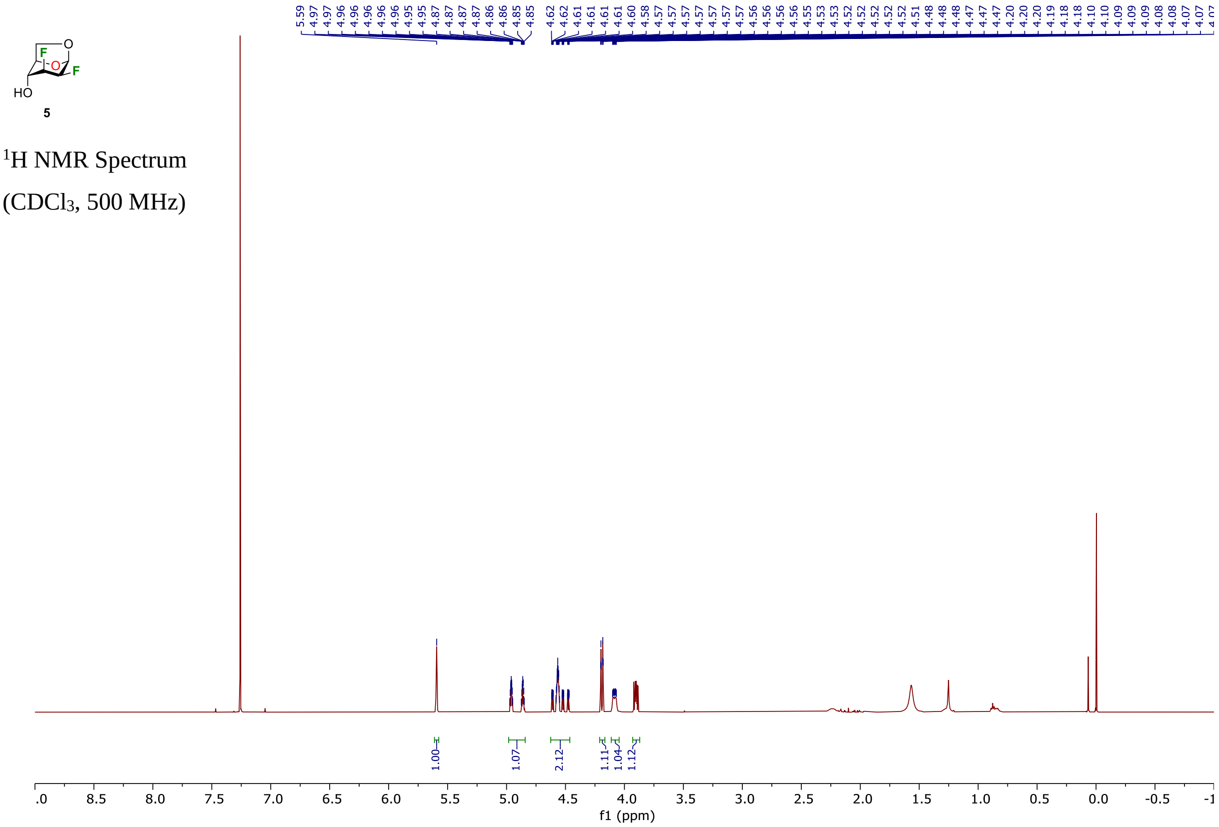
5
^{1}H NMR Spectrum
(CDCl$_3$, 500 MHz)
f1 (ppm)

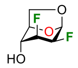

5

^{13}C NMR Spectrum

($CDCl_3$, 126 MHz)

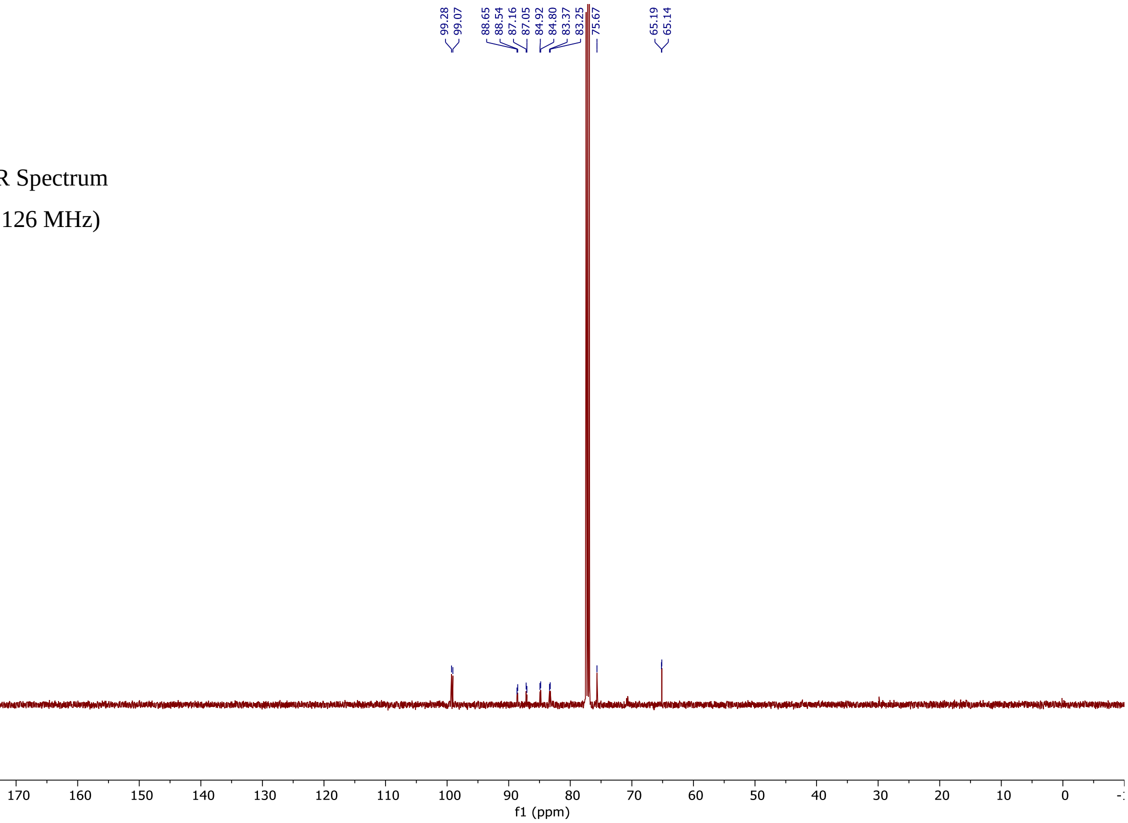

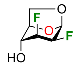

5

^{19}F NMR Spectrum

($CDCl_3$, 470 MHz)

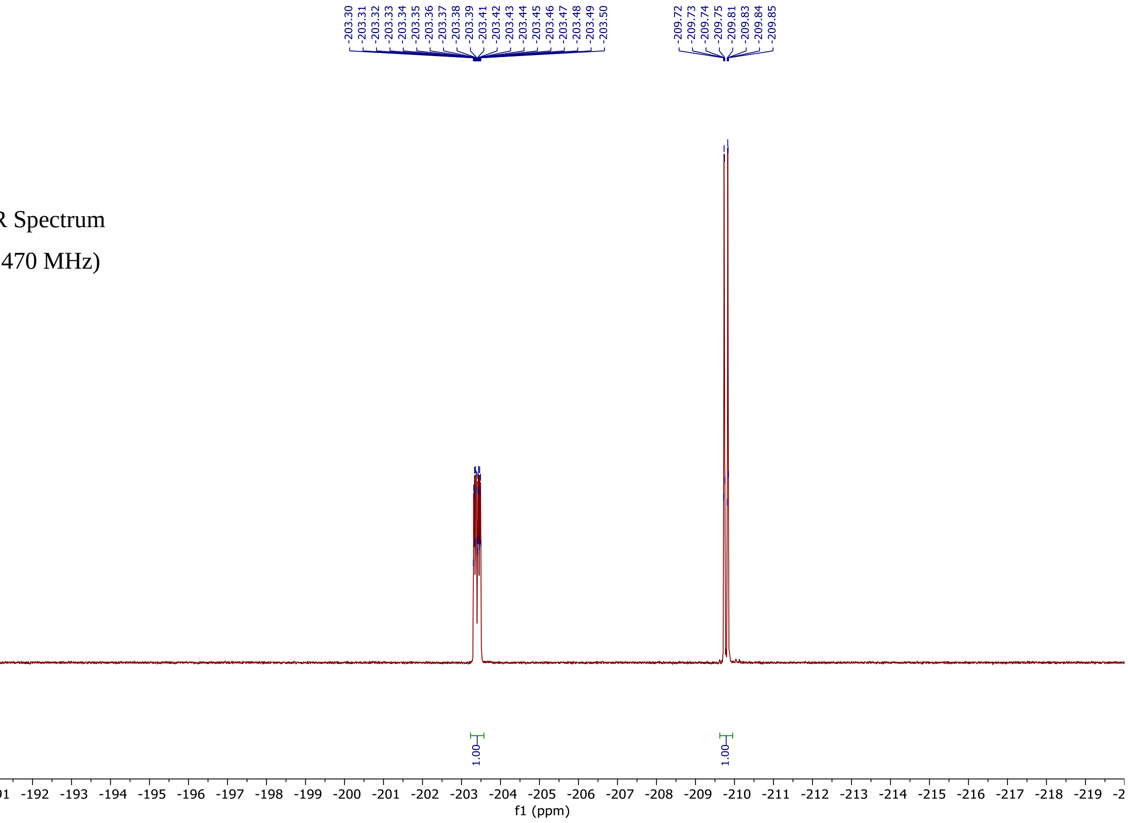

Compound 9

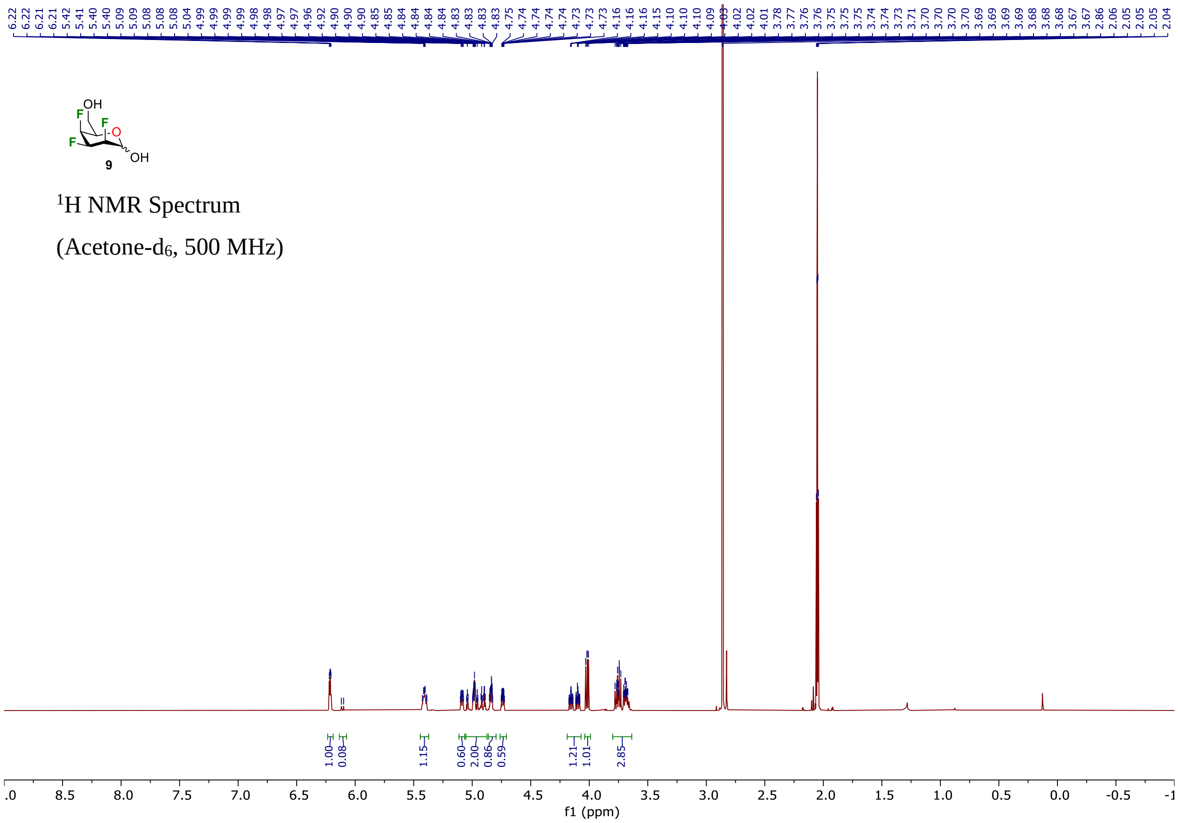

6.22 6.22 6.21 6.21 5.42 5.41 5.40 5.40 5.09 5.09 5.08 5.08 5.04 4.99 4.99 4.99 4.98 4.98 4.97 4.97 4.96 4.92 4.90 4.90 4.90 4.85 4.85 4.84 4.84 4.84 4.84 4.83 4.83 4.83 4.83 4.75 4.74 4.74 4.74 4.73 4.73 4.73 4.16 4.16 4.16 4.15 4.10 4.10 4.10 4.09 4.03 4.02 4.02 4.01 3.78 3.77 3.76 3.76 3.75 3.75 3.75 3.74 3.74 3.73 3.71 3.70 3.70 3.70 3.69 3.69 3.69 3.68 3.68 3.68 3.67 2.86 2.06 2.05 2.05 2.04
OH
F
F
O
F
OH
9
1H NMR Spectrum
(Acetone-d6, 500 MHz)
1.00
0.08
1.15
0.60
2.00
0.86
0.59
1.21
1.01
2.85
f1 (ppm)

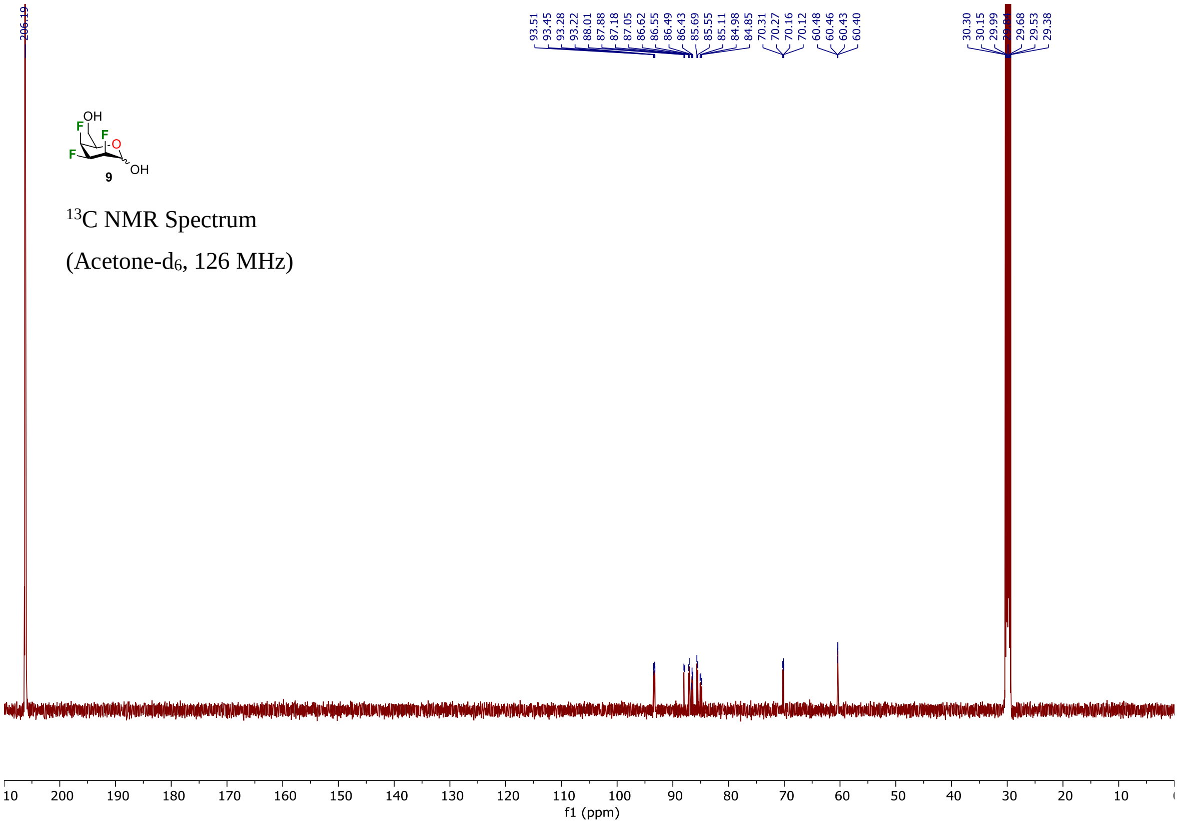

206.19
93.51 93.45 93.28 93.22 88.01 87.88 87.18 87.05 86.62 86.55 86.49 86.43 85.69 85.55 85.11 84.98 84.85 70.31 70.27 70.16 70.12 60.48 60.46 60.43 60.40
30.30 30.15 29.99 29.68 29.53 29.38
OH
F
F
O
F
OH
9
13C NMR Spectrum
(Acetone-d6, 126 MHz)
f1 (ppm)

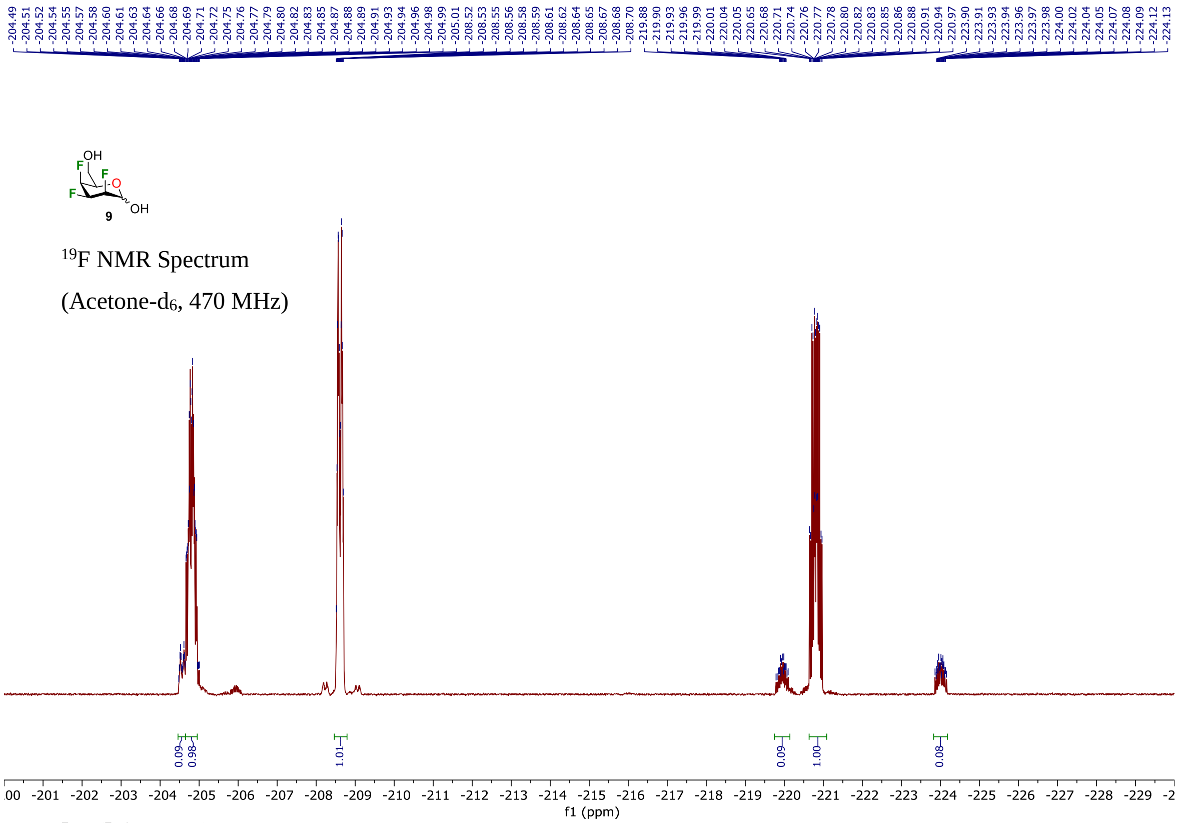

Compound 1(bisOTs)

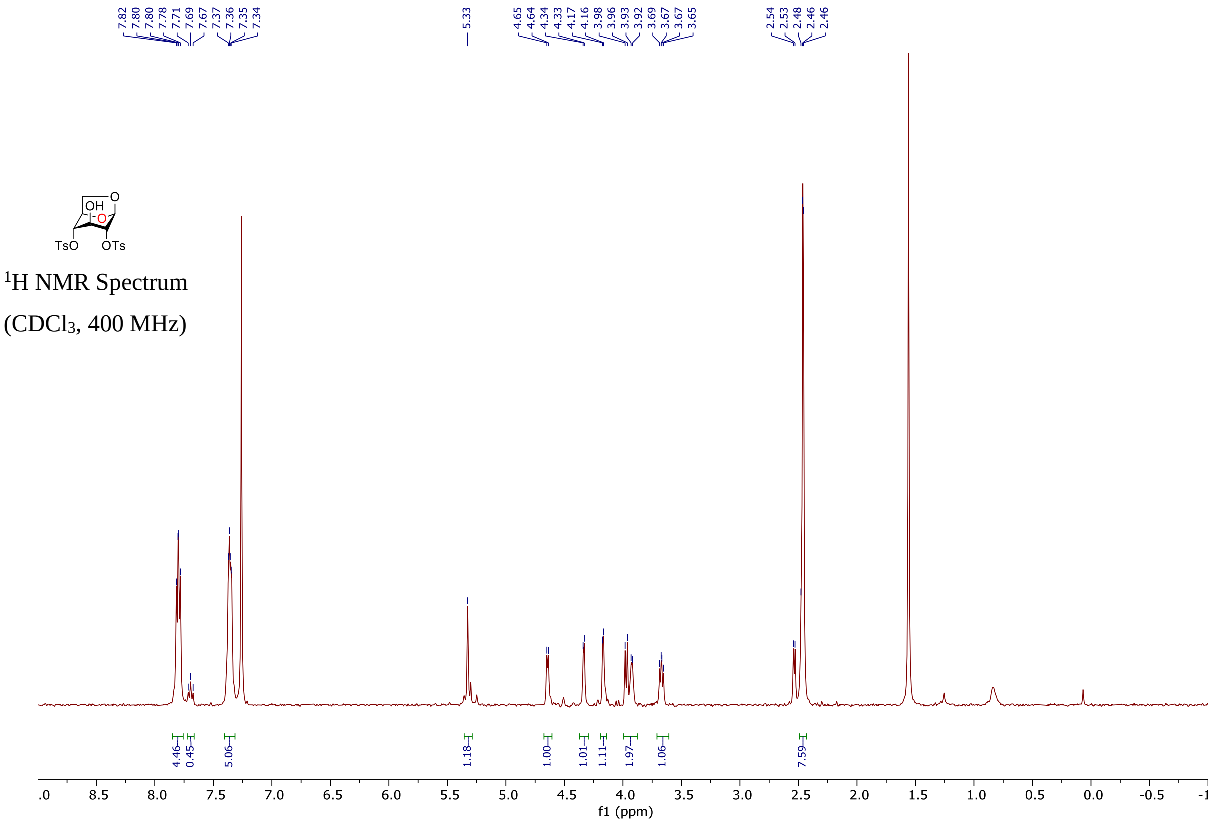

Compound 21(1,6-anhydro)

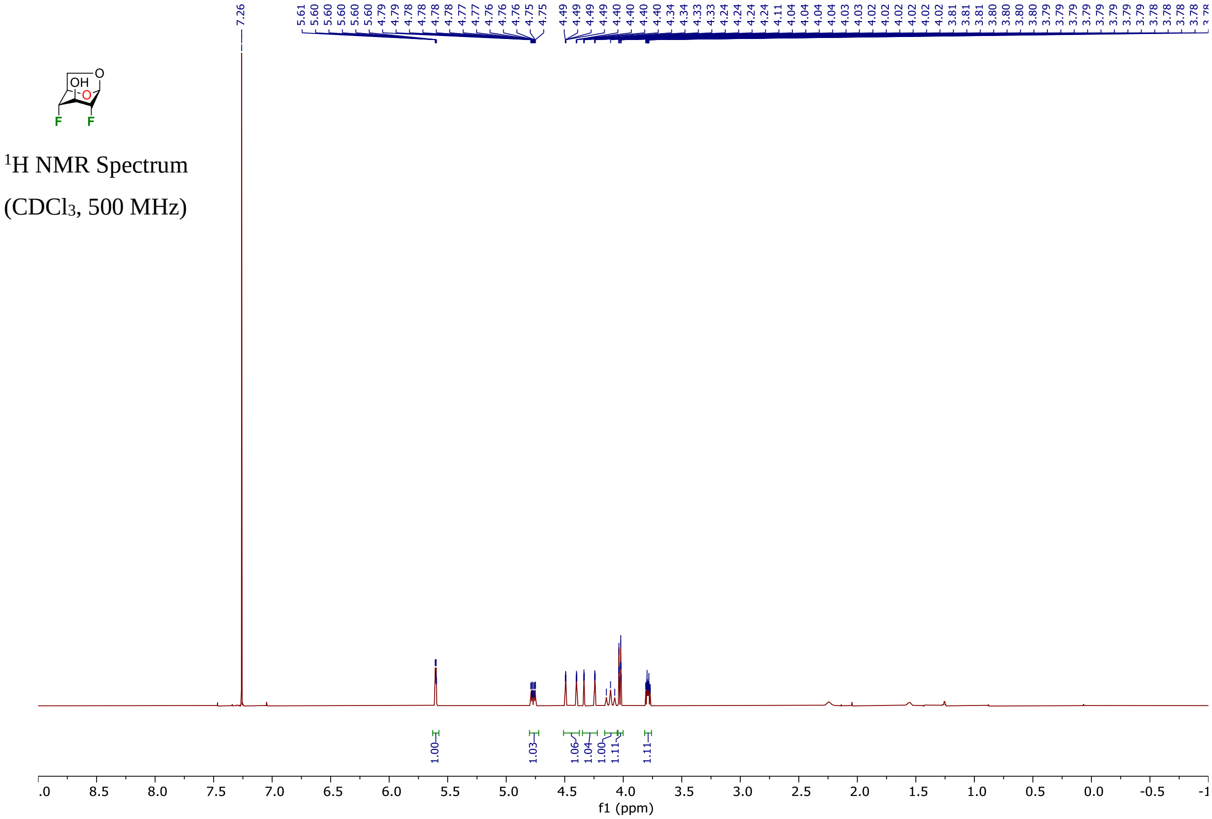

^{1}H NMR Spectrum

($CDCl_3$, 500 MHz)

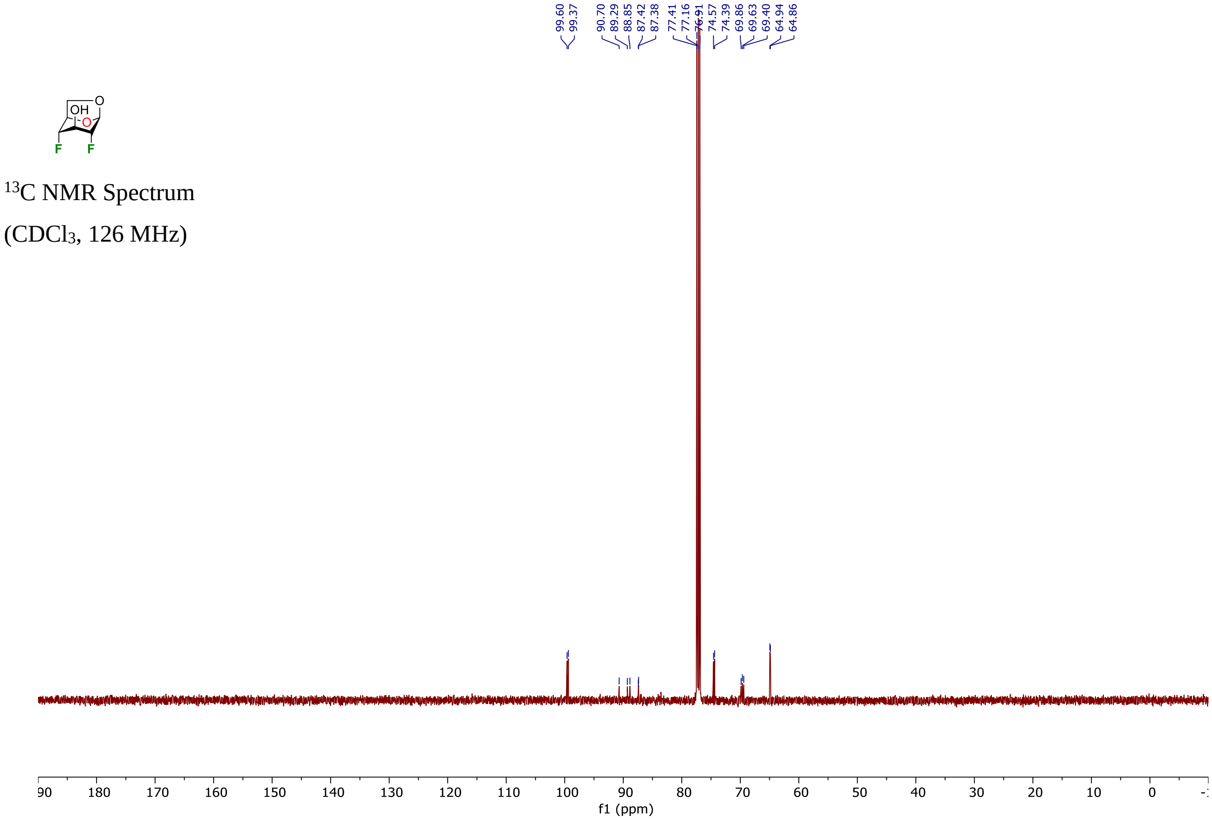

^{13}C NMR Spectrum

($CDCl_3$, 126 MHz)

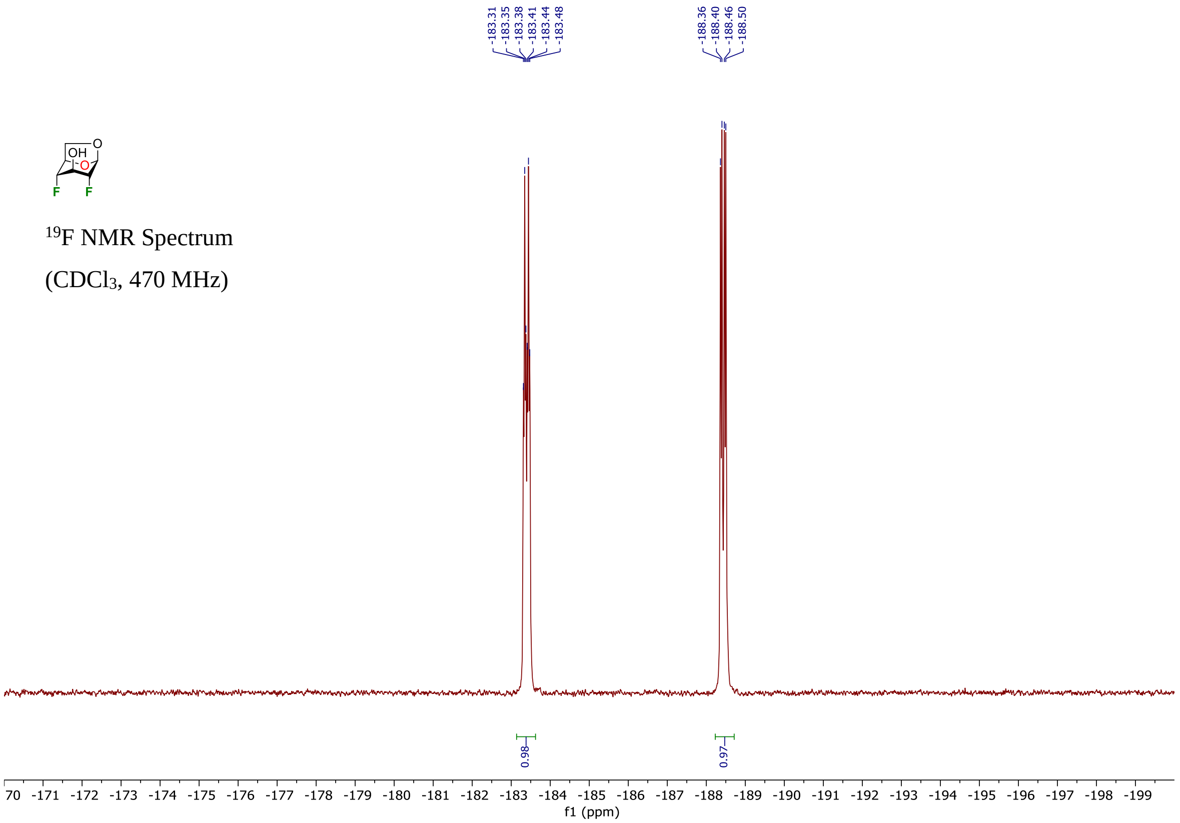
-183.31
-183.35
-183.38
-183.41
-183.44
-183.48
-188.36
-188.40
-188.46
-188.50
OH
O
O
F
F
^{19}F NMR Spectrum
(CDCl$_3$, 470 MHz)
0.98
0.97
70 -171 -172 -173 -174 -175 -176 -177 -178 -179 -180 -181 -182 -183 -184 -185 -186 -187 -188 -189 -190 -191 -192 -193 -194 -195 -196 -197 -198 -199
f1 (ppm)

Compound 21

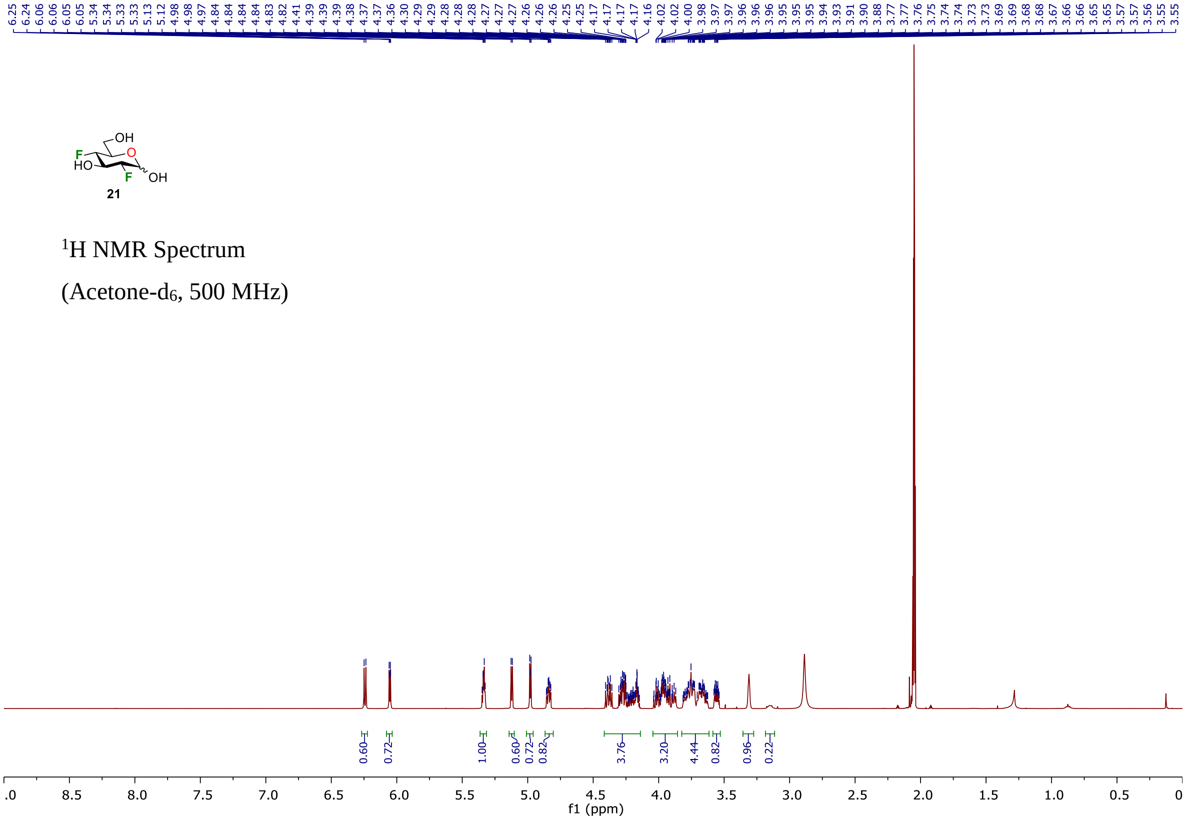
6.25 6.24 6.06 6.05 5.34 5.33 5.33 5.13 5.12 4.98 4.98 4.97 4.84 4.84 4.84 4.83 4.82 4.41 4.39 4.39 4.39 4.38 4.37 4.37 4.36 4.30 4.29 4.29 4.28 4.28 4.28 4.27 4.27 4.27 4.26 4.26 4.26 4.25 4.25 4.17 4.17 4.17 4.17 4.16 4.02 4.02 4.00 3.98 3.97 3.97 3.96 3.96 3.95 3.95 3.95 3.94 3.93 3.91 3.90 3.88 3.77 3.77 3.76 3.75 3.74 3.74 3.73 3.73 3.69 3.68 3.68 3.67 3.66 3.66 3.65 3.65 3.57 3.57 3.56 3.55 3.55
OH
F
HO
O
F
OH
21
^{1}H NMR Spectrum
(Acetone-d$_6$, 500 MHz)
0.60
0.72
1.00
0.60
0.72
0.82
3.76
3.20
4.44
0.82
0.96
0.22
.0 8.5 8.0 7.5 7.0 6.5 6.0 5.5 5.0 4.5 4.0 3.5 3.0 2.5 2.0 1.5 1.0 0.5 0
f1 (ppm)

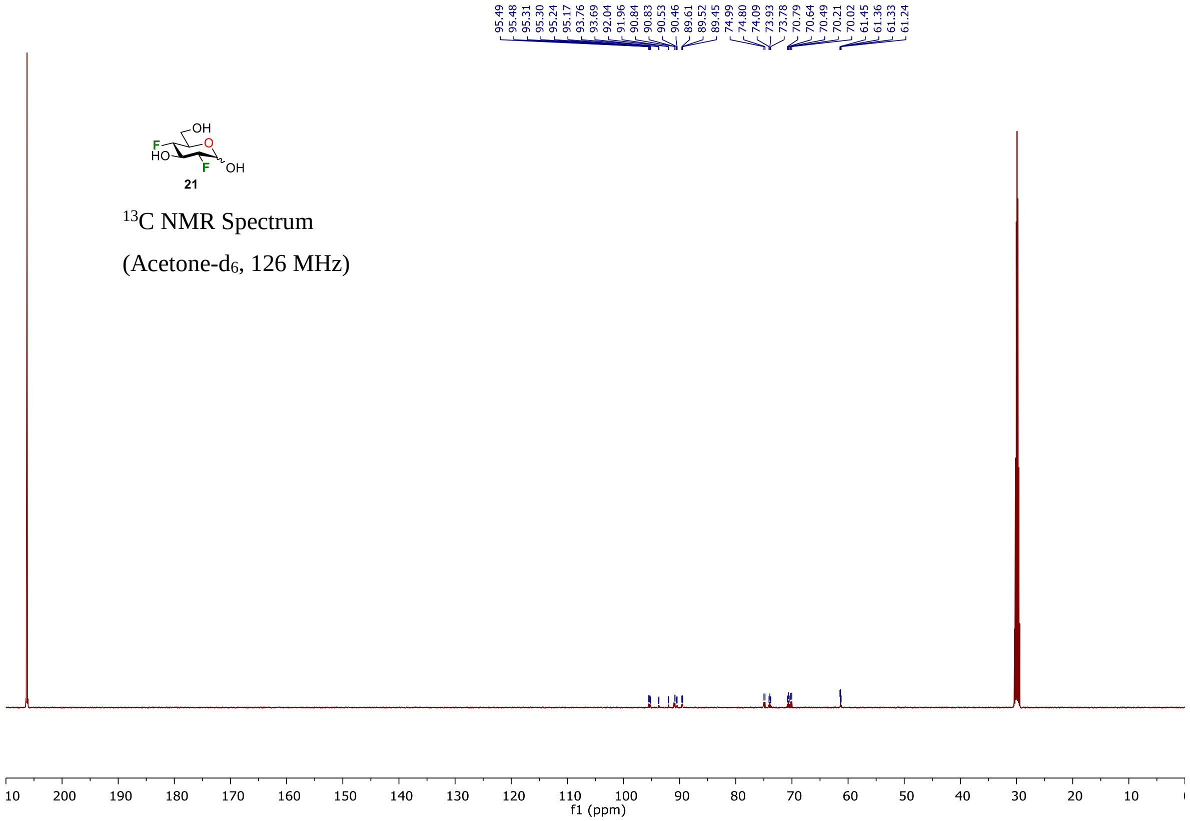
95.49 95.48 95.31 95.30 95.24 95.17 93.76 93.69 92.04 91.96 90.84 90.83 90.53 90.46 89.61 89.52 89.45 74.99 74.80 74.09 73.93 73.78 70.79 70.64 70.49 70.21 70.02 61.45 61.36 61.33 61.24
OH
F
O
HO
F
OH
21
^{13}C NMR Spectrum
(Acetone-d$_6$, 126 MHz)
10 200 190 180 170 160 150 140 130 120 110 100 90 80 70 60 50 40 30 20 10
f1 (ppm)

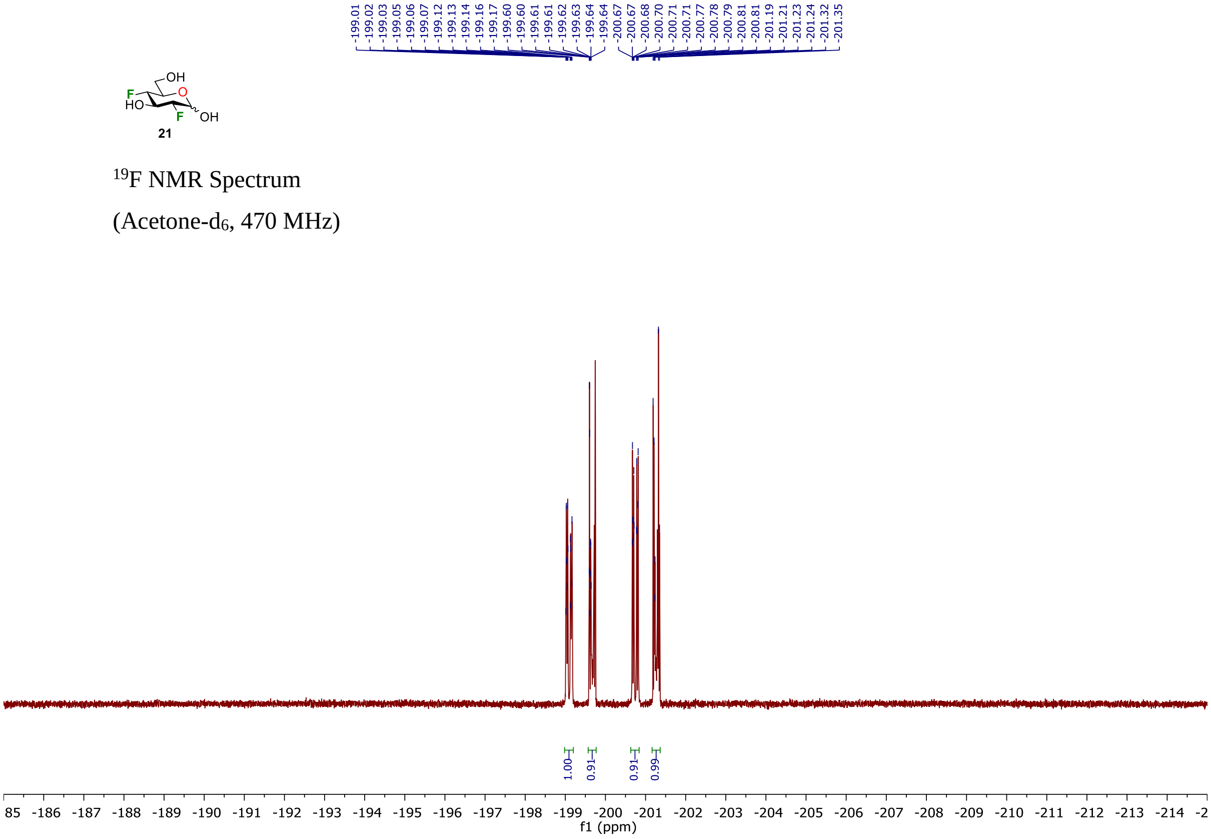
-199.01 -199.02 -199.03 -199.05 -199.06 -199.07 -199.12 -199.13 -199.14 -199.16 -199.17 -199.60 -199.60 -199.61 -199.61 -199.62 -199.63 -199.64 -199.64 -200.67 -200.68 -200.70 -200.71 -200.71 -200.77 -200.78 -200.79 -200.81 -200.81 -201.19 -201.21 -201.23 -201.24 -201.32 -201.35
OH
F
O
HO
F
OH
21
^{19}F NMR Spectrum
(Acetone-d$_6$, 470 MHz)
1.00 0.91 0.91 0.99
85 -186 -187 -188 -189 -190 -191 -192 -193 -194 -195 -196 -197 -198 -199 -200 -201 -202 -203 -204 -205 -206 -207 -208 -209 -210 -211 -212 -213 -214 -2
f1 (ppm)

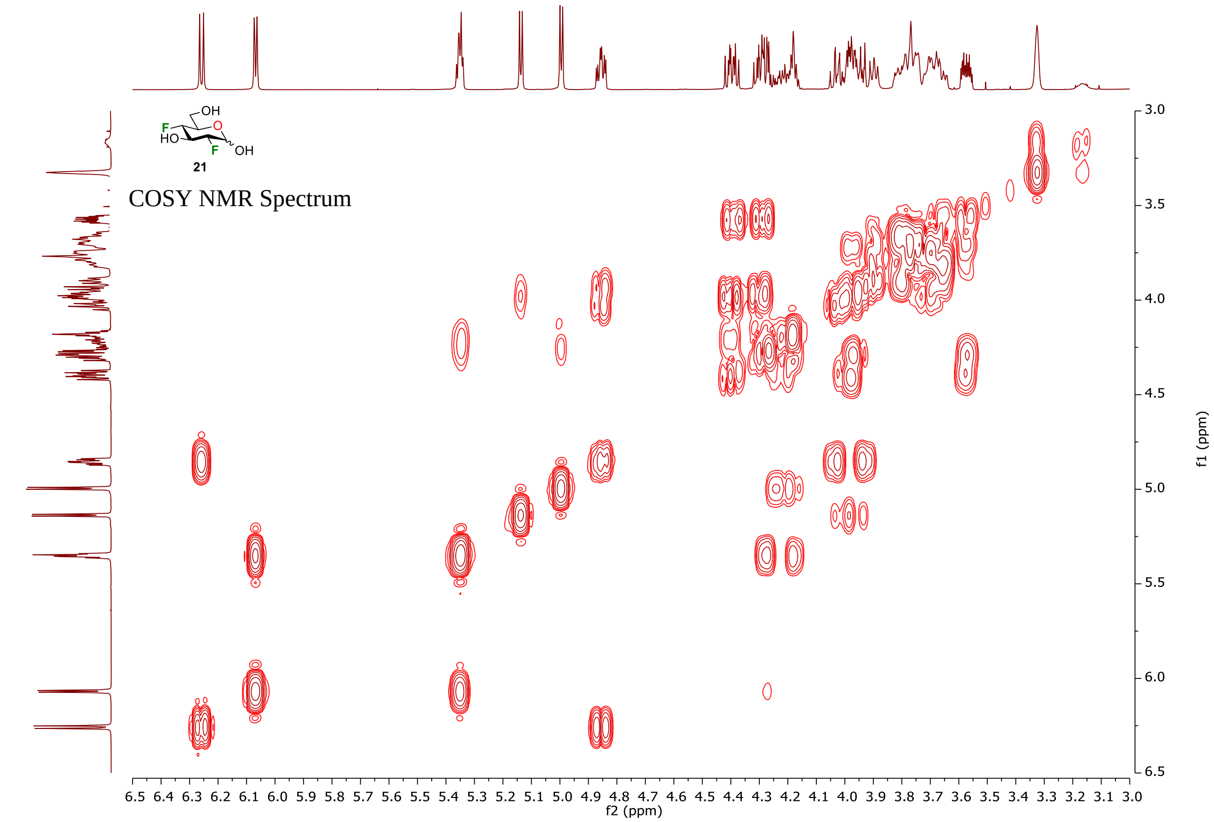
OH
F
HO
O
F
OH
21
COSY NMR Spectrum
f1 (ppm)
f2 (ppm)

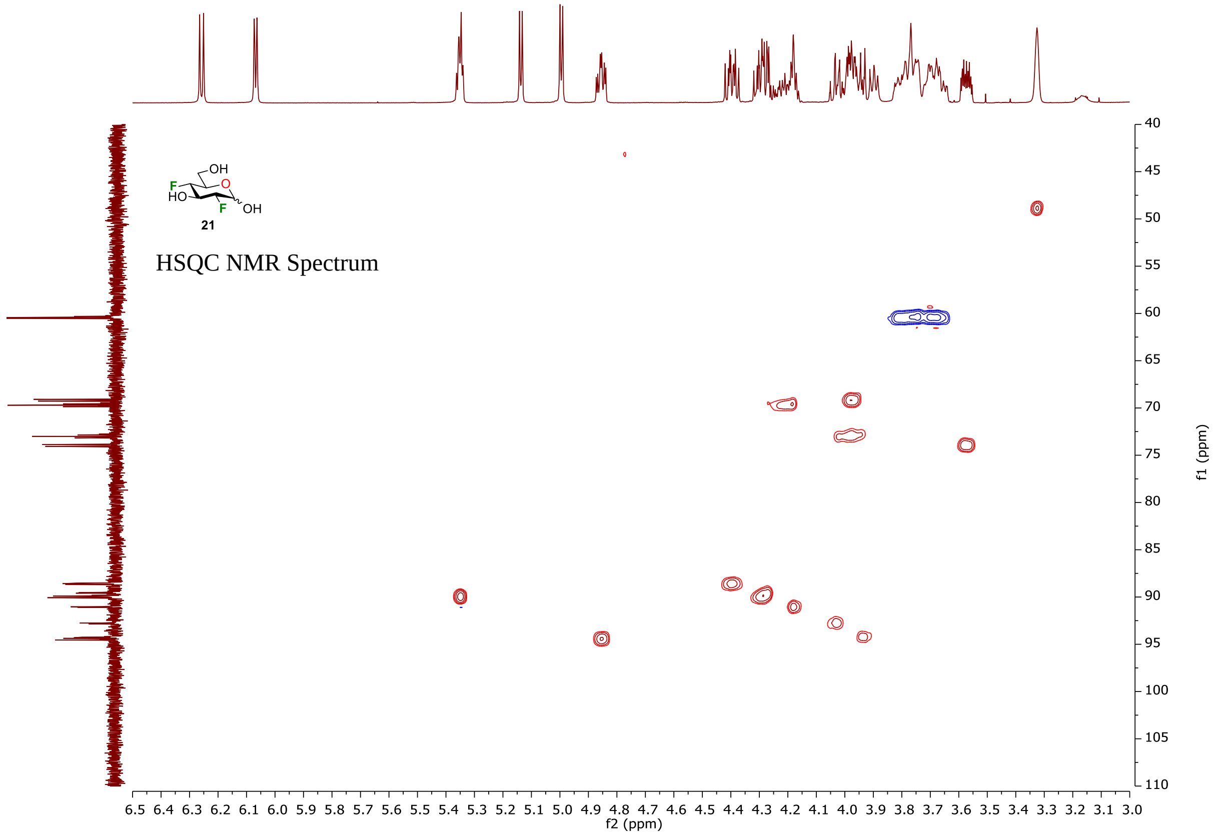
OH
F
HO
O
F
OH
21
HSQC NMR Spectrum
f1 (ppm)
f2 (ppm)

Compound 22

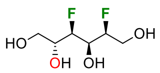

22

^{1}H NMR Spectrum

(Acetone-d_6, 500 MHz)

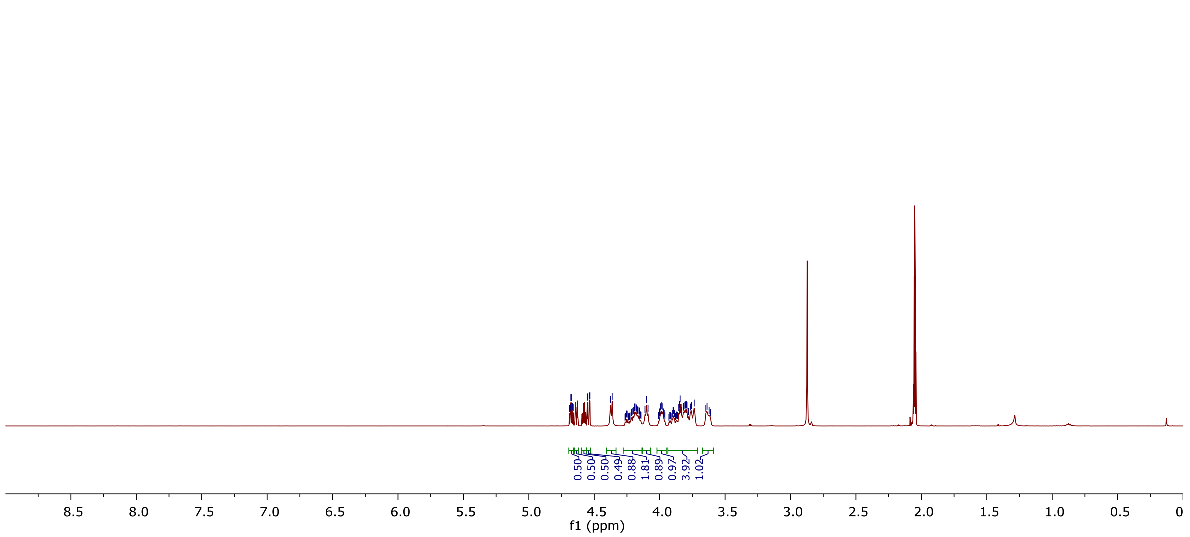

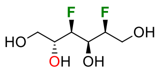

22

^{13}C NMR Spectrum

(Acetone-d_6, 126 MHz)

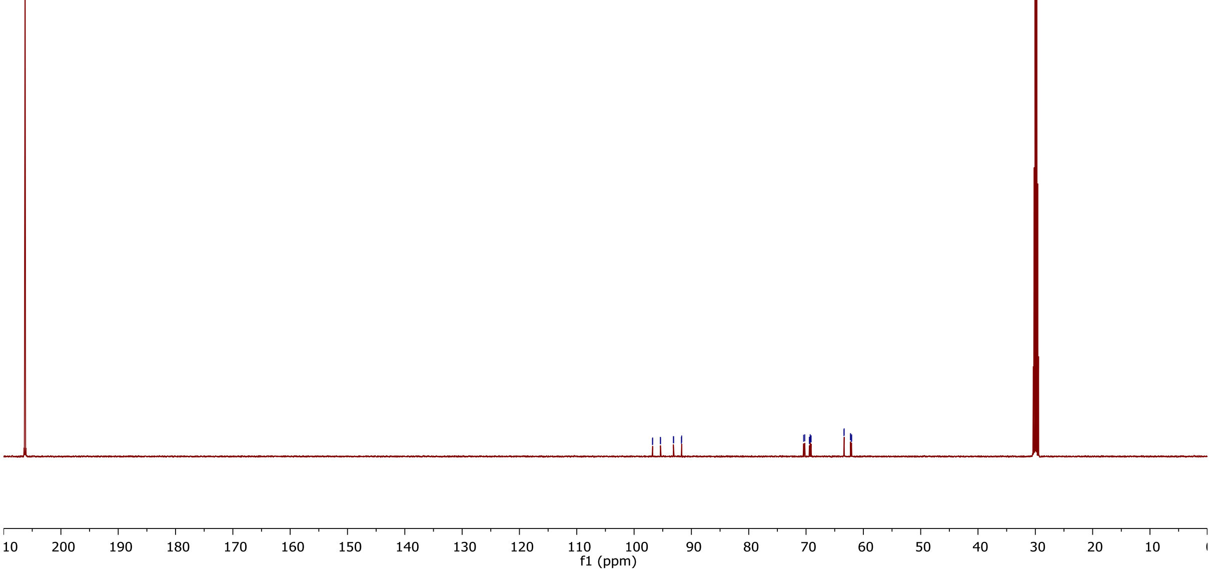

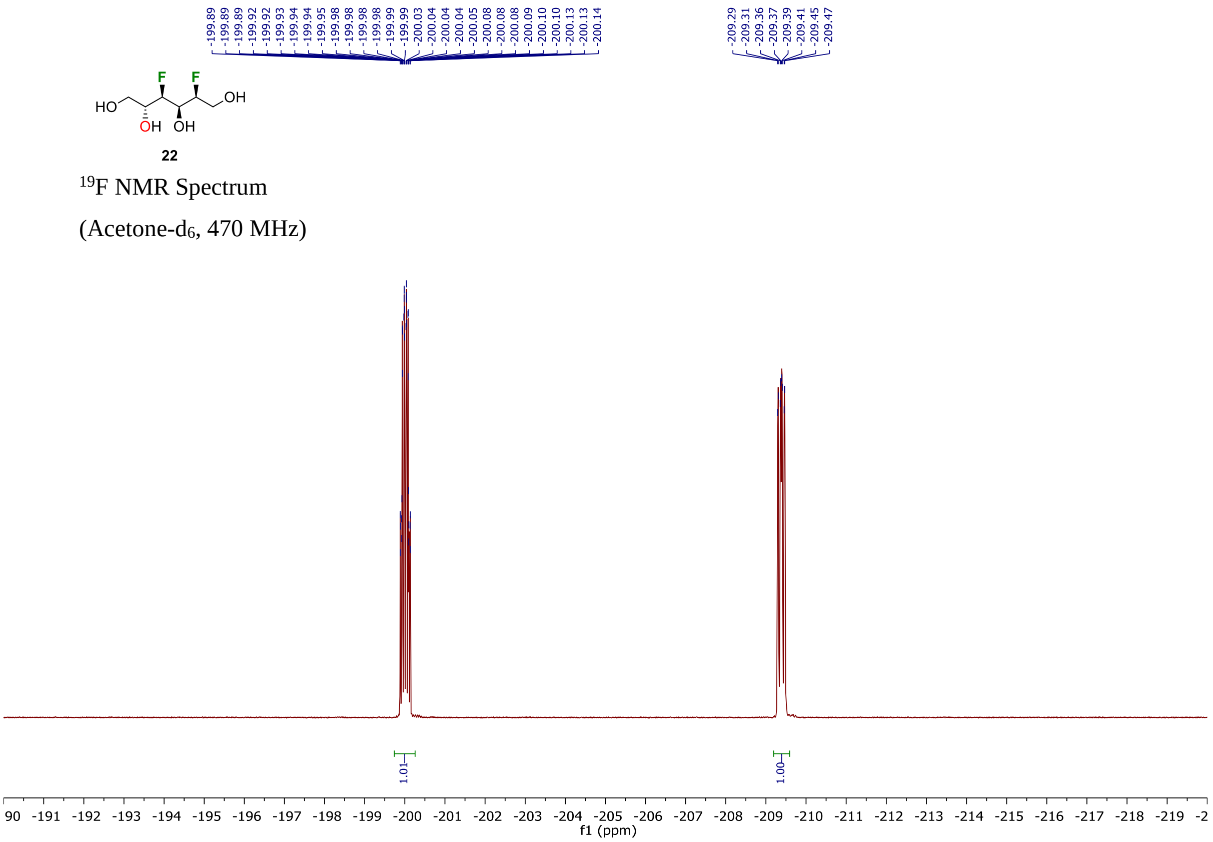
-199.89
-199.89
-199.89
-199.92
-199.92
-199.93
-199.94
-199.94
-199.95
-199.98
-199.98
-199.98
-199.98
-199.99
-199.99
-200.03
-200.04
-200.04
-200.04
-200.05
-200.08
-200.08
-200.08
-200.09
-200.10
-200.10
-200.13
-200.13
-200.14
-209.29
-209.31
-209.36
-209.37
-209.39
-209.41
-209.45
-209.47
22
^{19}F NMR Spectrum
(Acetone-d_6, 470 MHz)
1.01
1.00
f1 (ppm)

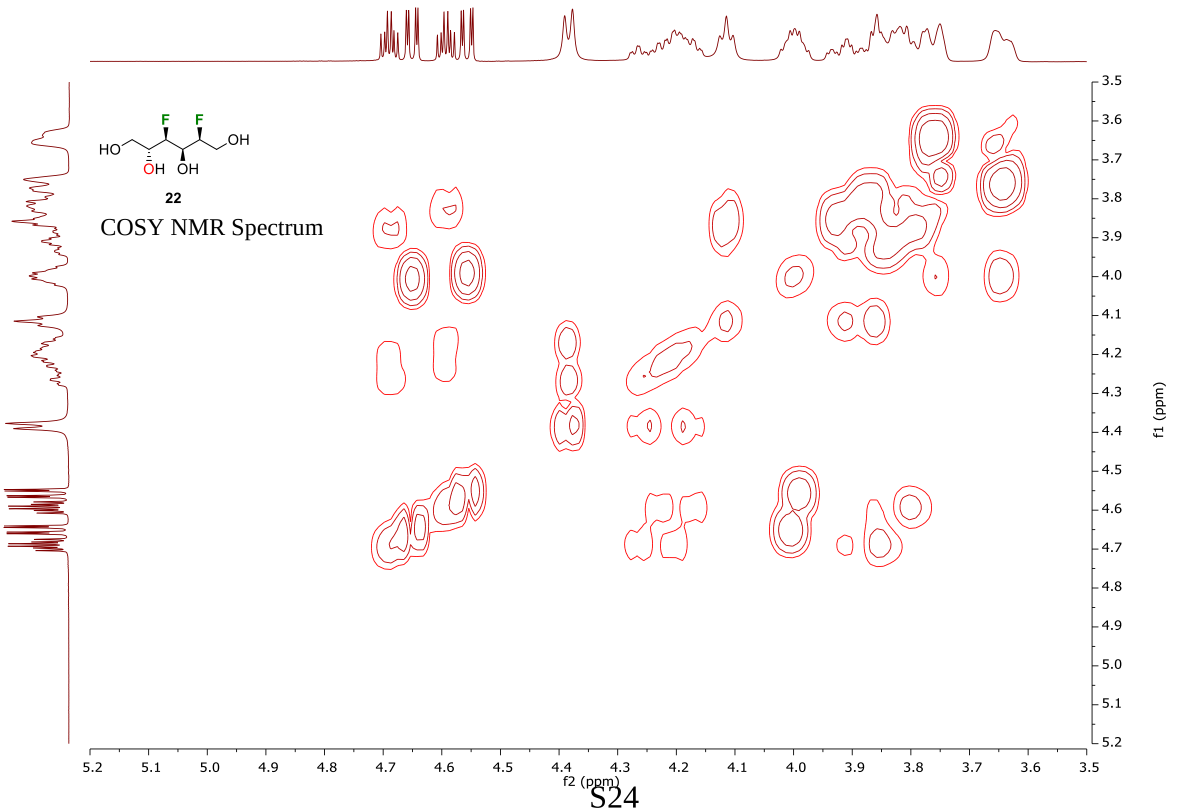
22
COSY NMR Spectrum
f2 (ppm)
f1 (ppm)

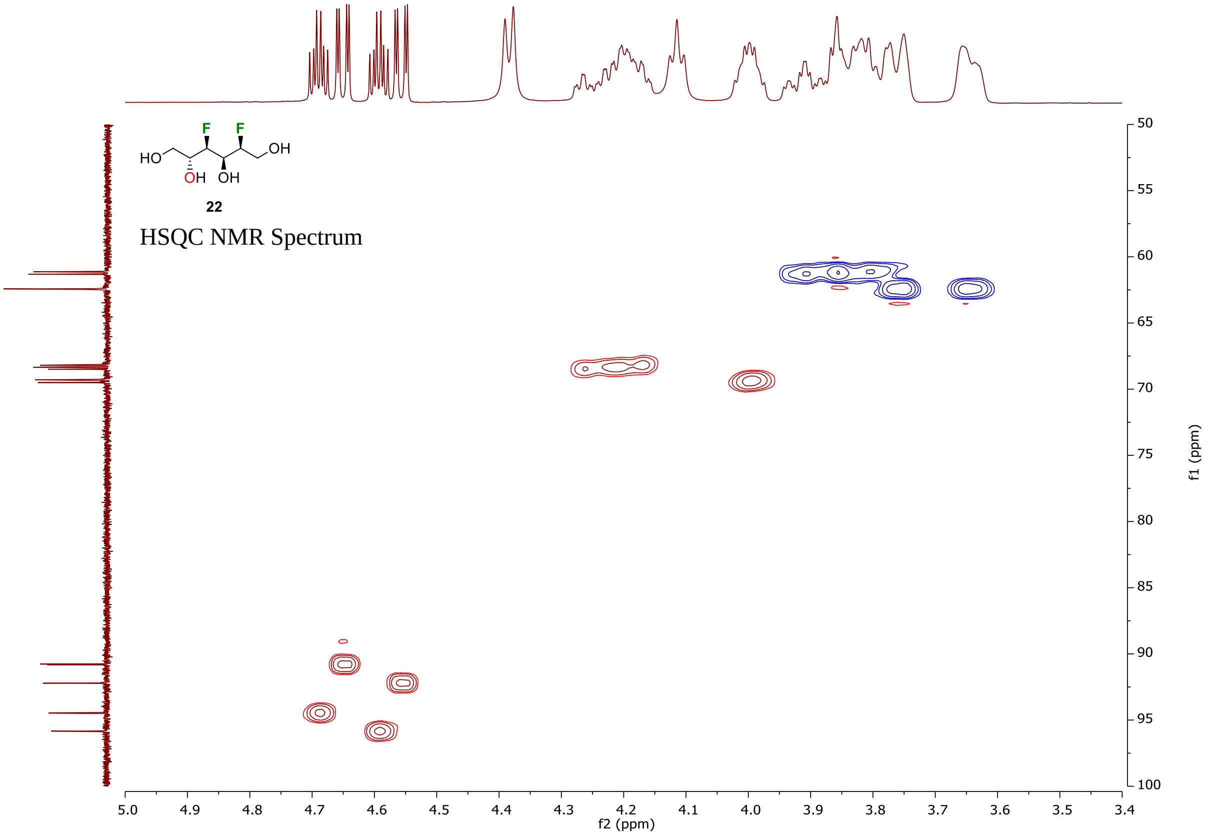
HO
OH
OH
F
F
OH
22
HSQC NMR Spectrum
f1 (ppm)
f2 (ppm)

Compound 23

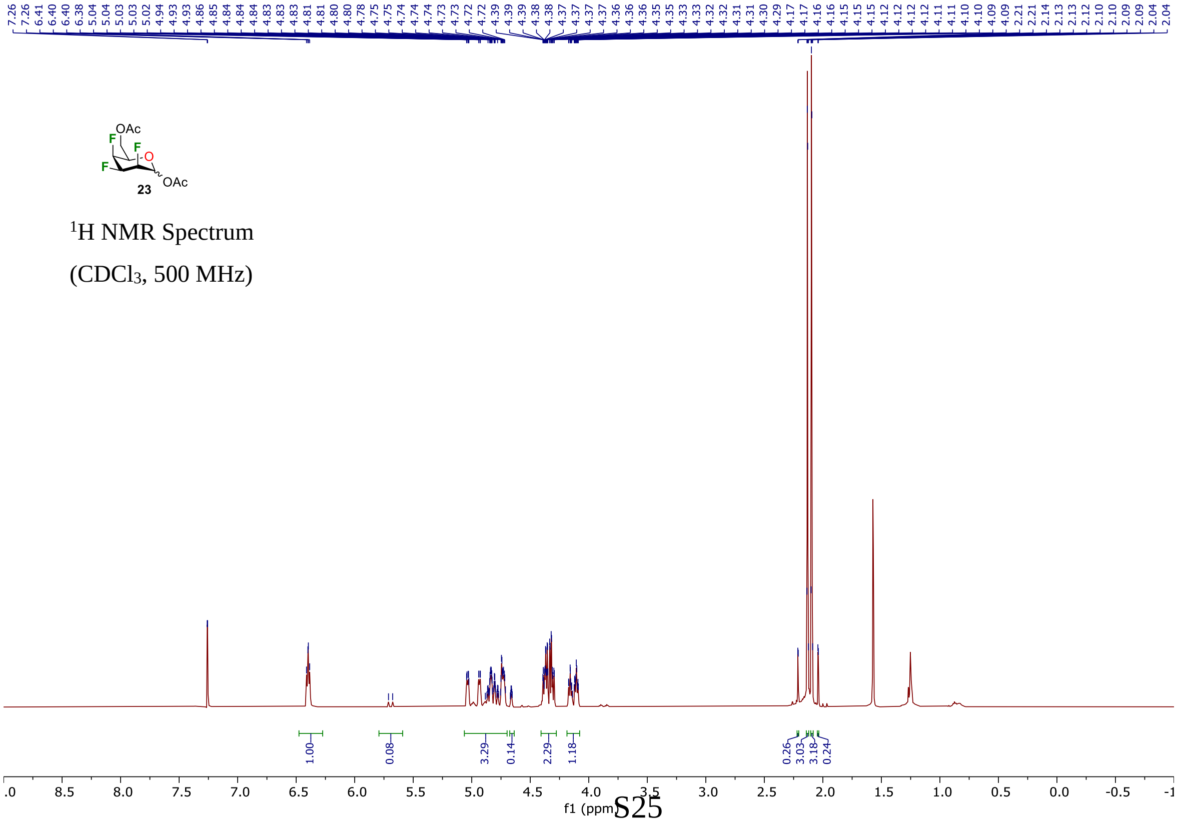
OAc
F
F
O
F
OAc
23
^{1}H NMR Spectrum
(CDCl3, 500 MHz)
1.00
0.08
3.29
0.14
2.29
1.18
0.26
3.03
3.18
0.24
f1 (ppm)

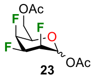

^{13}C NMR Spectrum

($CDCl_3$, 126 MHz)

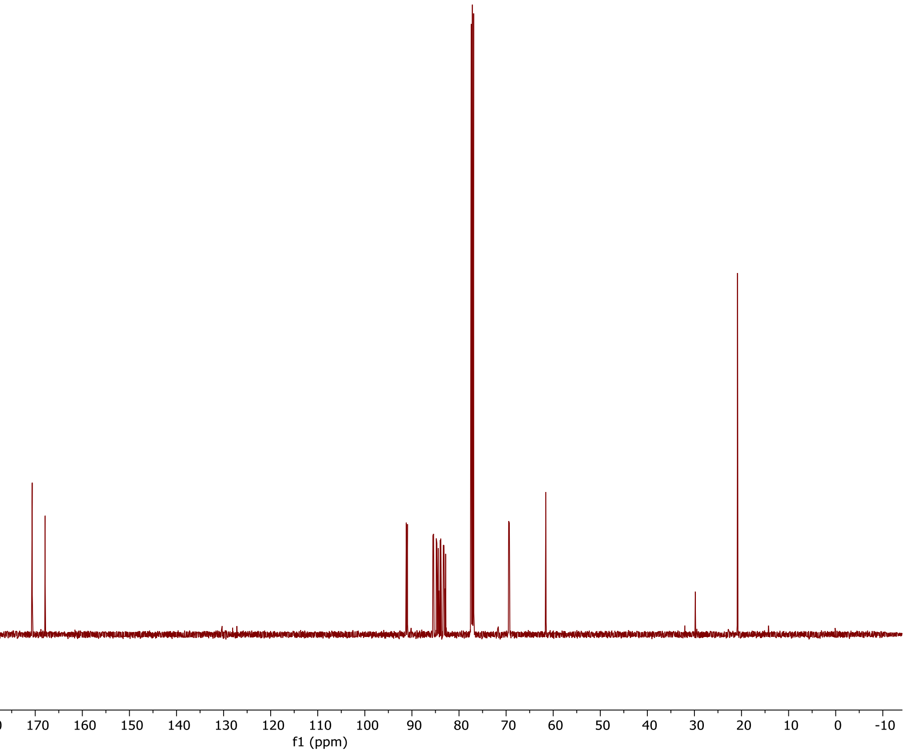

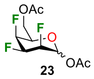

^{19}F NMR Spectrum

($CDCl_3$, 470 MHz)

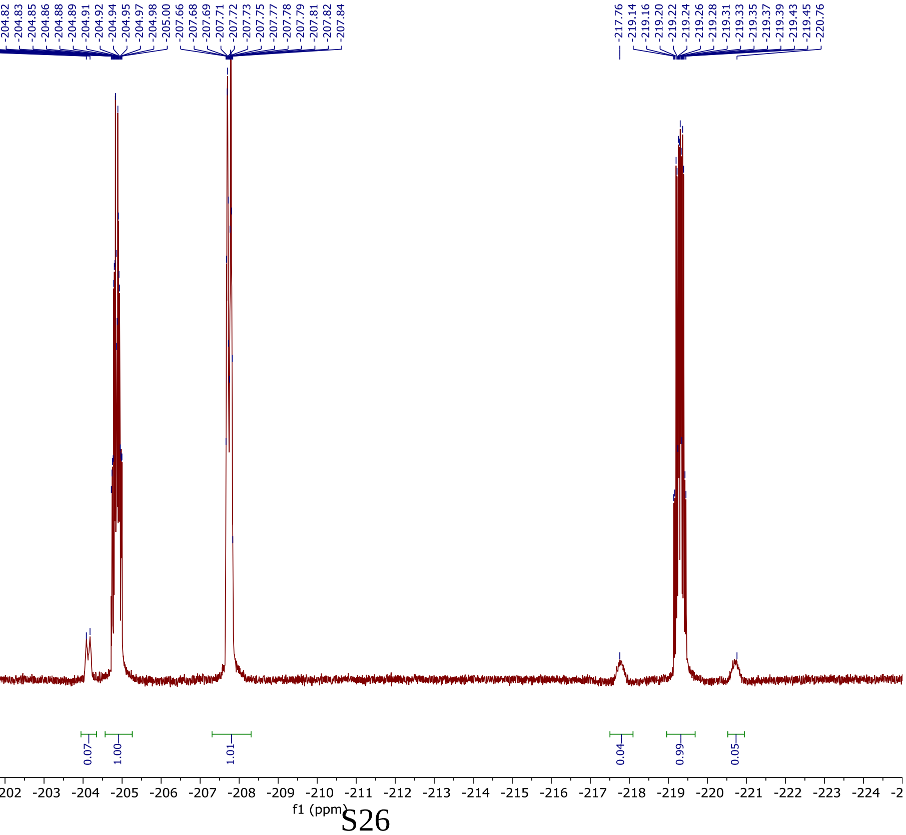

Compound 24

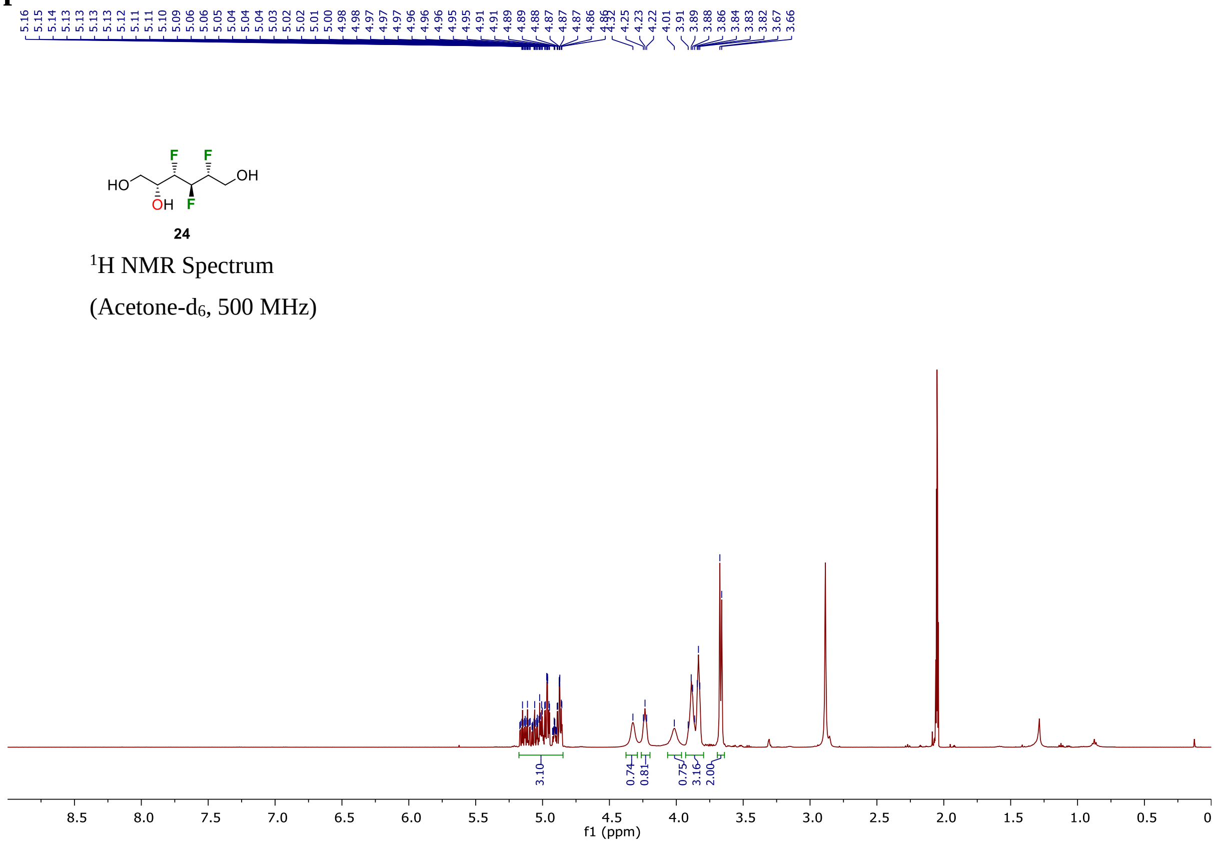
5.16 5.15 5.14 5.13 5.13 5.13 5.13 5.12 5.11 5.11 5.10 5.09 5.06 5.06 5.05 5.04 5.04 5.04 5.03 5.02 5.02 5.01 5.00 4.98 4.98 4.97 4.97 4.97 4.96 4.96 4.96 4.95 4.95 4.91 4.91 4.89 4.89 4.88 4.87 4.87 4.87 4.86 4.86 4.32 4.25 4.23 4.22 4.01 3.91 3.89 3.88 3.86 3.84 3.83 3.82 3.67 3.66
HO
OH
OH
F
F
F
24
1H NMR Spectrum
(Acetone-d6, 500 MHz)
3.10
0.74
0.81
0.75
3.16
2.00
f1 (ppm)

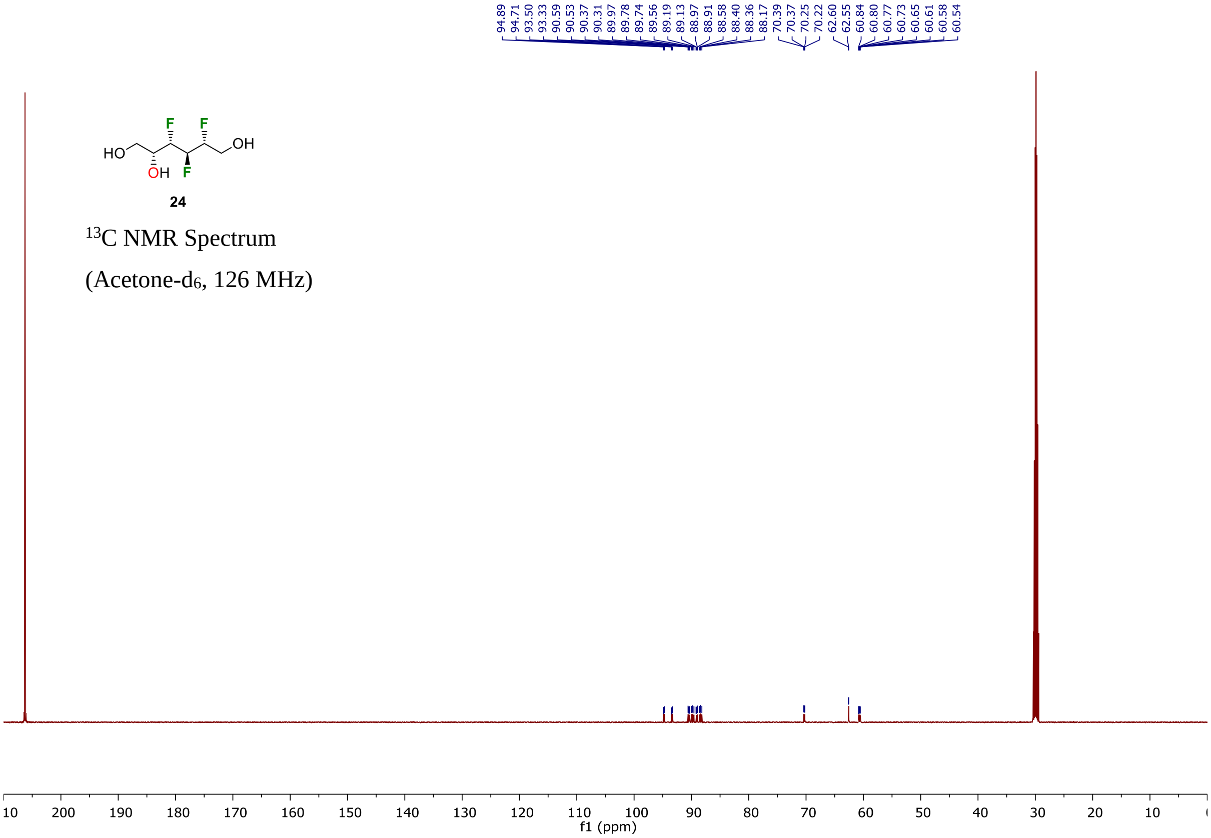
94.89 94.71 93.50 93.33 90.59 90.53 90.37 90.31 89.97 89.78 89.74 89.56 89.19 89.13 88.97 88.91 88.58 88.40 88.36 88.17 70.39 70.37 70.25 70.22 62.60 62.55 60.84 60.80 60.77 60.73 60.65 60.61 60.58 60.54
HO
OH
OH
F
F
F
24
13C NMR Spectrum
(Acetone-d6, 126 MHz)
f1 (ppm)

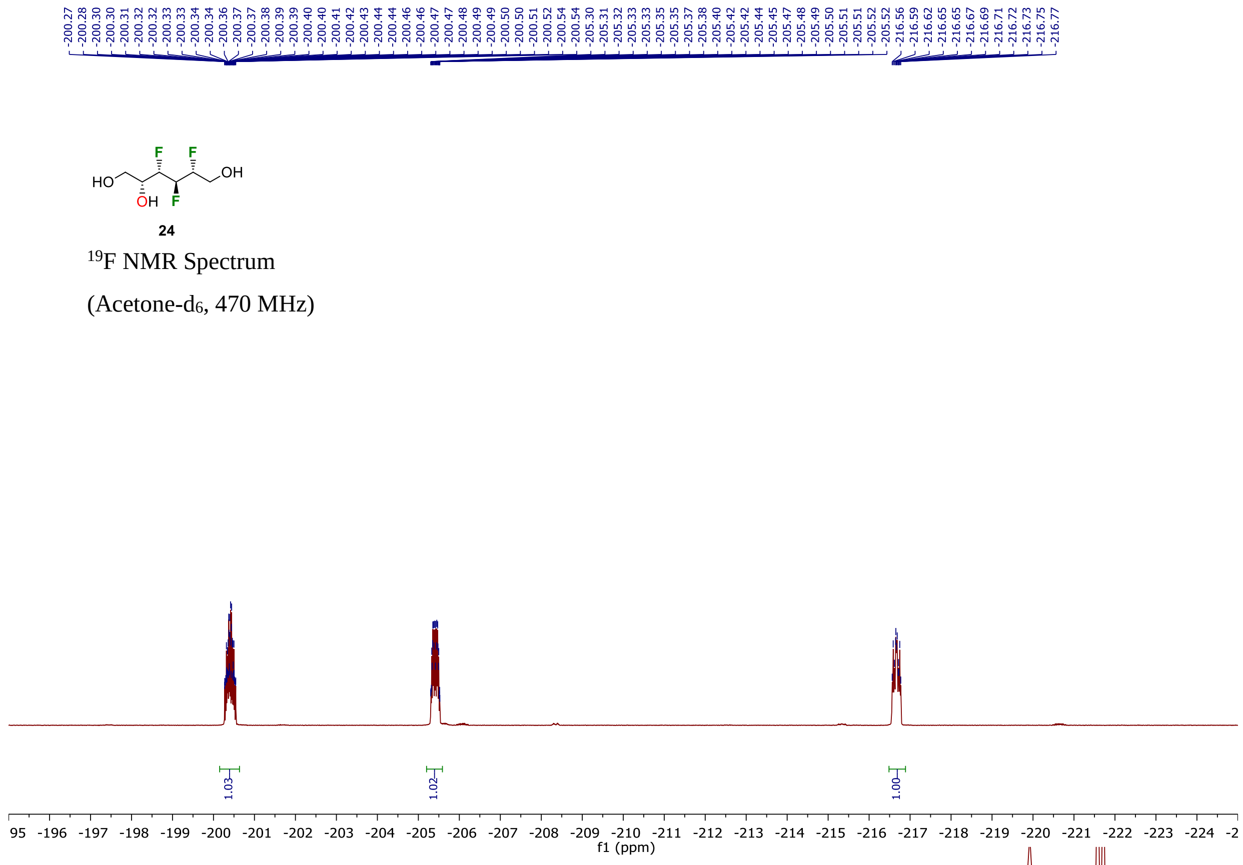

24

^{19}F NMR Spectrum

(Acetone-d_6, 470 MHz)

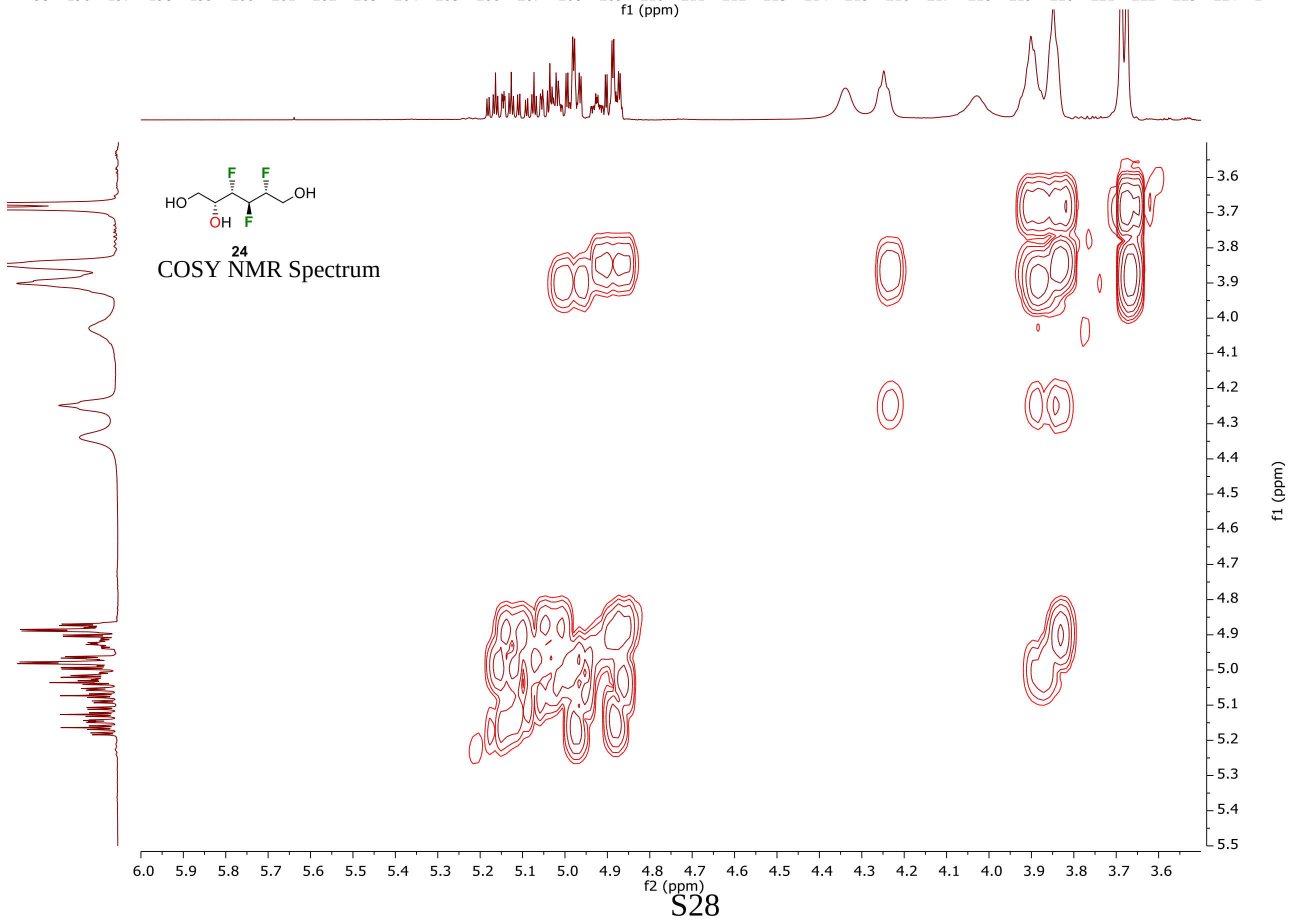

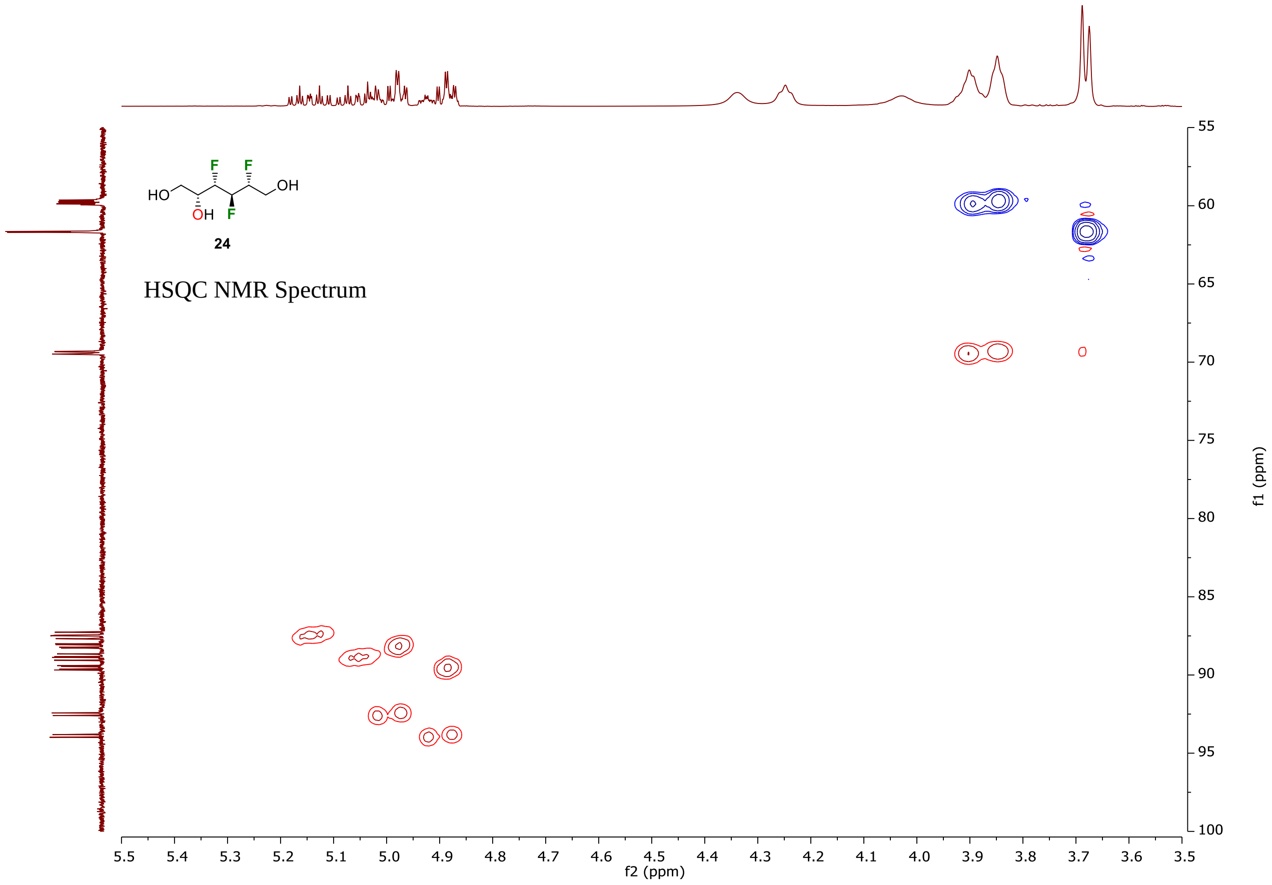
HO
OH
F
F
OH
F
24
HSQC NMR Spectrum
f1 (ppm)
f2 (ppm)

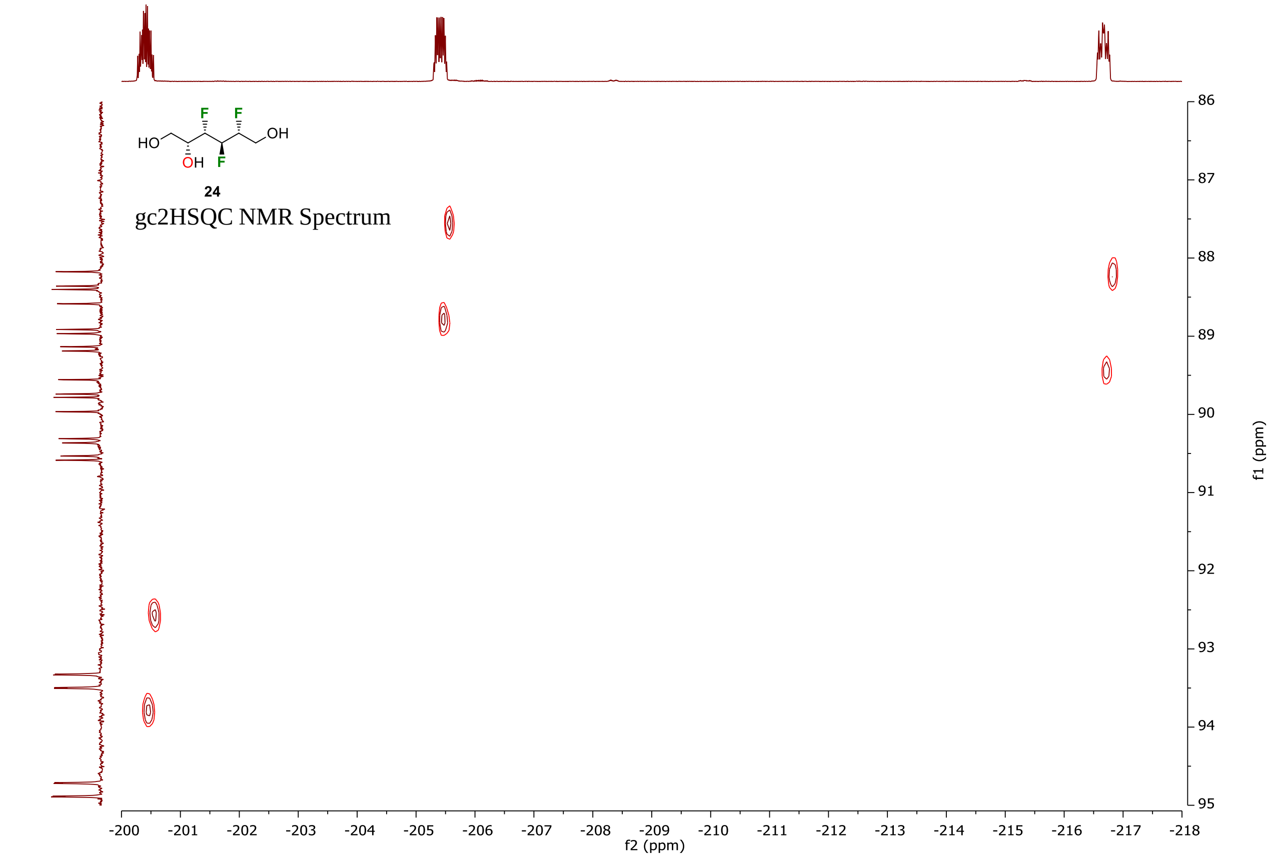
HO
OH
F
F
OH
F
24
gc2HSQC NMR Spectrum
f1 (ppm)
f2 (ppm)

IV. References

[1] St-Gelais, J.; Côté, É.; Lainé, D.; Johnson, P. A.; Giguère, D. *Chem. Eur. J.* **2020**, *26*, 13499−13506.

[2] St-Gelais, J.; Bouchard, M.; Denavit, V.; Giguère, D. *J. Org. Chem.* **2019**, *84*, 8509–8522.

[3] Denavit, V.; Lainé, D.; St-Gelais, J.; Johnson, P. A.; Giguère, D. *Nat. Commun.* **2018**, *9*, 4721.
